# Canonical Notch signaling controls the early thymic epithelial progenitor cell state and emergence of the medullary epithelial lineage in fetal thymus development

**DOI:** 10.1242/dev.178582

**Published:** 2020-06-22

**Authors:** Dong Liu, Anastasia I. Kousa, Kathy E. O'Neill, Paul Rouse, Martyna Popis, Alison M. Farley, Simon R. Tomlinson, Svetlana Ulyanchenko, Francois Guillemot, Philip A. Seymour, Mette C. Jørgensen, Palle Serup, Ute Koch, Freddy Radtke, C. Clare Blackburn

**Affiliations:** 1 MRC Centre for Regenerative Medicine, Institute for Stem Cell Research, School of Biological Sciences, 5, Little France Drive, Edinburgh EH16 4UU, UK; 2 Biotech Research and Innovation Centre (BRIC), Ole Maaløes Vej 5, 2200 Copenhagen, Denmark; 3 The Francis Crick Institute, 1 Midland Road, London NW1 1AT, UK; 4 NNF Center for Stem Cell Biology, University of Copenhagen, Nørre Alle 14, DK-2200 Copenhagen N, Denmark; 5 Ecole Polytechnique Fédérale de Lausanne (EPFL), 1015 Lausanne, Switzerland

**Keywords:** Thymus, Thymic epithelial cell, Stem cell, Progenitor cell, Lineage divergence, Differentiation, Cell fate regulation, Notch signaling

## Abstract

Thymus function depends on the epithelial compartment of the thymic stroma. Cortical thymic epithelial cells (cTECs) regulate T cell lineage commitment and positive selection, while medullary (m) TECs impose central tolerance on the T cell repertoire. During thymus organogenesis, these functionally distinct sub-lineages are thought to arise from a common thymic epithelial progenitor cell (TEPC). However, the mechanisms controlling cTEC and mTEC production from the common TEPC are not understood. Here, we show that emergence of the earliest mTEC lineage-restricted progenitors requires active NOTCH signaling in progenitor TEC and that, once specified, further mTEC development is NOTCH independent. In addition, we demonstrate that persistent NOTCH activity favors maintenance of undifferentiated TEPCs at the expense of cTEC differentiation. Finally, we uncover a cross-regulatory relationship between NOTCH and FOXN1, a master regulator of TEC differentiation. These data establish NOTCH as a potent regulator of TEPC and mTEC fate during fetal thymus development, and are thus of high relevance to strategies aimed at generating/regenerating functional thymic tissue *in vitro* and *in vivo*.

## Abstract (precis)

**Summary:** Notch signaling regulates the initial emergence of medullary thymic epithelial sublineage, implicating Notch in the maintenance of primitive thymic epithelial progenitors and uncovering its cross-interaction with Foxn1.

## INTRODUCTION

In the thymus, thymic epithelial cells (TECs) are the essential stromal component required for T lymphocyte development (Manley et al., 2011; Ritter and Boyd, 1993). Two functionally distinct TEC subsets, cortical (c) TECs and medullary (m) TECs, exist and are found in the cortex and the medulla of the organ, respectively. Thymocytes migrate in a highly stereotypical fashion to encounter cTECs and mTECs sequentially as T cell differentiation and repertoire selection proceeds (Anderson and Takahama, 2012; Klein et al., 2014). Broadly, cortical thymic epithelial cells (cTECs) regulate T cell lineage commitment and positive selection, while medullary (m) TECs impose central tolerance on the T cell repertoire (Abramson and Anderson, 2017). The crucial role for mTEC in tolerance induction depends on expression of autoimmune regulator (AIRE), which regulates promiscuous expression of numerous otherwise tissue-restricted genes, and on AIRE-independent mechanisms that may in part be regulated by FEZF2 (Abramson and Anderson, 2017; Anderson and Su, 2016; Anderson et al., 2002; Fujikado et al., 2016; Kyewski and Peterson, 2010; Takaba et al., 2015; Yang et al., 2015).

cTECs and mTECs originate from endodermal progenitor cells (thymic epithelial progenitor cells; TEPCs) that are present in the thymic primordium during its initial generation from the third pharyngeal pouches (3PPs) (Gordon et al., 2004; Le Douarin and Jotereau, 1975; Rossi et al., 2006). Several studies have shown that, during development, both cTECs and mTECs arise from cells expressing markers associated with mature cTECs, including CD205 and β5t (Baik et al., 2013; Ohigashi et al., 2013), while clonal analyses have shown that a bipotent TEPC can exist *in vivo* (Bleul et al., 2006; Rossi et al., 2006). Based on these observations, a serial progression model of TEC differentiation has been proposed (Alves et al., 2014). This suggests that fetal TEPCs exhibit features associated with the cTEC lineage and that additional cues are required for mTEC specification from this common TEPC. Identification of cTEC-restricted sub-lineage specific progenitor TECs in the fetal thymus has proved elusive, owing to the shared expression of surface antigens between this presumptive cell type and the presumptive common TEPC (Alves et al., 2014; Baik et al., 2013; Shakib et al., 2009), although cTEC-restricted progenitors clearly exist in the postnatal thymus (Ulyanchenko et al., 2016). In contrast, the presence of mTEC-restricted progenitors has been detected from day 13.5 of embryonic development (E13.5) (Rodewald et al., 2001). In the fetal thymus, these mTEC progenitors are characterized by expression of claudins 3 and 4 (CLDN3/4), and SSEA1 (Hamazaki et al., 2007; Sekai et al., 2014). Receptors leading to activation of the nuclear factor kappa-light-chain-enhancer of activated B cells (NF-κB) pathway, including lymphotoxin-β receptor (LTβR) and receptor activator of NF-κB (RANK), are known to regulate the proliferation and maturation of mTEC through crosstalk with T cells and lymphoid tissue inducer cells (Boehm et al., 2003; Hikosaka et al., 2008; Rossi et al., 2007); recently, a hierarchy of intermediate progenitors specific for the mTEC sublineage has been proposed based on genetic analysis of NF-κB pathway components (Akiyama et al., 2016; Baik et al., 2016). Additionally, histone deacetylase 3 (HDAC3) has emerged as an essential regulator of mTEC differentiation (Goldfarb et al., 2016), and a role for signal transducer and activator of transcription 3 (STAT3) signaling has been demonstrated in mTEC expansion and maintenance (Lomada et al., 2016; Satoh et al., 2016). Despite these advances, the molecular mechanisms governing the emergence of the earliest cTEC- and mTEC-restricted cells in thymic organogenesis are not yet understood (Hamazaki et al., 2007).

NOTCH signaling has been extensively studied in the context of thymocyte development (Shah and Zúñiga-Pflücker, 2014), and is also implicated as a regulator of TECs. Mice lacking the Notch ligand JAGGED 2 showed reduced medullary areas (Jiang et al., 1998), while B cells overexpressing another Notch ligand, Delta like 1 (DLL1), induced organized medullary areas in a reaggregate fetal thymic organ culture (RFTOC) system (Masuda et al., 2009). In contrast, in adult thymic epithelium NOTCH activity appeared to reside in a minor subpopulation of cTECs, while its TEC-specific overexpression reduced TEC cellularity and led to an imbalance between mature and immature mTECs, suggesting that NOTCH signaling might inhibit mTEC lineage development (Goldfarb et al., 2016). Overall, these results suggest that NOTCH has complex effects in TECs, but the stage(s) at and mechanism(s) through which NOTCH influences TEC development have not yet been determined.

We have addressed the role of NOTCH signaling in early TEC differentiation using loss- and gain-of-function analyses. Our data establish, via genetic ablation of NOTCH signaling in TECs using *Foxn1^Cre^;Rbpj^fl/fl^* and *Foxa2^Cre^;dnMAML* mice, and via fetal thymic organ culture (FTOC) in the presence of a NOTCH inhibitor, that NOTCH signaling is required for the initial emergence of mTEC lineage cells, and that NOTCH is required earlier than RANK-mediated signaling in mTEC development. They further show that NOTCH signaling is permissive, rather than instructive, for mTEC specification, as TEC-specific overexpression of the Notch intracellular domain (NICD) in fetal TEC dictated an undifferentiated TEPC phenotype rather than uniform adoption of mTEC characteristics. Finally, they uncover a cross-regulatory relationship between NOTCH and FOXN1, the master regulator of TEC differentiation. Collectively, our data establish NOTCH as a potent regulator of TEPC and mTEC fate during fetal thymus development.

## RESULTS

### Early fetal mTECs exhibit high NOTCH activity

**Fig. 1.**
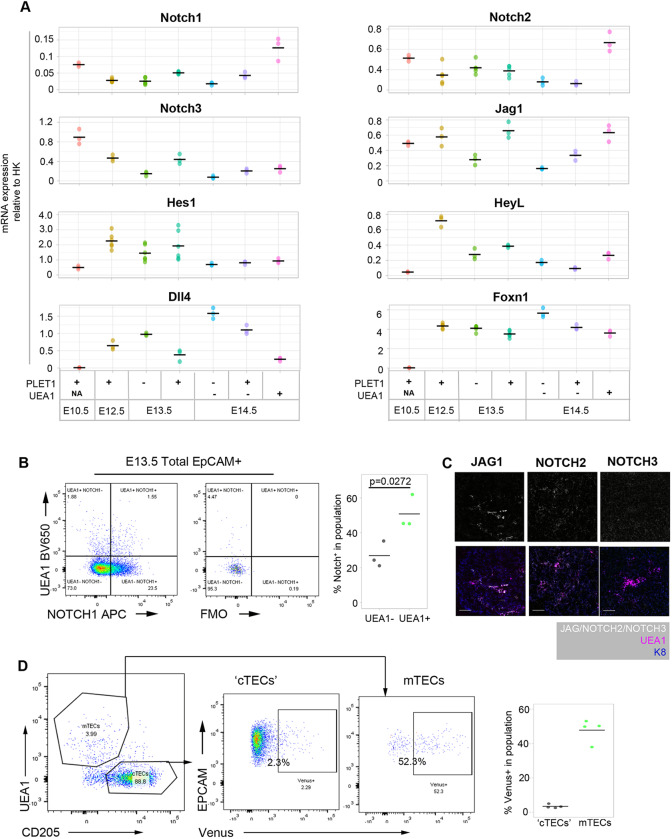
**Expression of Notch pathway components in thymus organogenesis.** (A) Plots show RT-qPCR analysis of Notch receptor, ligand and target expression from E10.5 to E14.5 in cell populations of the phenotypes shown. (B) Representative flow cytometry plots of Notch1 expression in E13.5 TECs, split by expression of UEA1. (C) Single images of JAG1, Notch2 and Notch3, and co-staining with the mTEC marker UEA1 and epithelial marker K8 on sections of E14.5 thymus primordium. Scale bars: 50 μm. (D) Left: representative profile of E14.5 CBF1:H2B-Venus thymi, gated on EPCAM^+^ epithelial cells. Cell suspension was stained with the mTEC marker UEA1 and the cTEC/progenitor (‘cTEC’) marker CD205. Middle: proportion of ‘cTECs’ and mTECs showing the expression of Venus. Right: quantitation of the percentage of Venus expression in E14.5 ‘cTEC’ and mTEC populations. (A) *n*=3 (all genes at E10.5 and E14.5, *Notch 3*, *Jag1*, *Heyl*, *Dll4* at E12.5 and E13.5) or 6 (*Notch 1*, *Notch 2*, *Hes1* and *Foxn1* at E12.5 and E13.5). In each case, *n* represents RNA obtained from pooled cells of the phenotype stated from an independent litter of embryos. All data points are shown. (B) Plots shown are representative of *n*=3. Each ‘*n*’ represents cells obtained from pooled thymi from an individual wild-type litter. (C) *n*=3 independent immunohistochemistry analyses. (D) *n*=4. Each ‘*n*’ is an independent E14.5 embryo from the same CBF1:Venus×C57BL6 litter; genotypes were retrospectively confirmed. *P* value in B was calculated using an unpaired two-tailed *t*-test.

To begin to understand how NOTCH signaling affects thymus development, we first investigated the expression of NOTCH ligands and receptors in TECs during early organogenesis, via RT-qPCR of E10.5 3PP cells and defined E12.5 to E14.5 TEC populations separated on the basis of EPCAM (which marks TECs), PLET1 (which marks the founder cells of the thymic epithelial lineage, is progressively downregulated with differentiation in most fetal TECs, and is also expressed in some postnatal mTECs and in a minor TEC progenitor subpopulation able to generate cTECs and mTECs upon transplantation; Depreter et al., 2008; Nowell et al., 2011; Ulyanchenko et al., 2016) and UEA1 (which marks mTECs) expression as appropriate (Fig. 1; for gating strategies see Fig. S1).

*Notch1*, *Notch2*, *Notch3*, *Jagged 1* (*Jag1*) and *Delta like 4 (Dll4*), but no other Notch receptors and ligands, were expressed throughout this time period (Fig. 1). *Notch1* and *Notch2* were significantly enriched in E14.5 UEA1^+^ mTECs compared with all other populations examined. *Notch3* and *Jag1* were more highly expressed in PLET1^+^ and UEA1^+^ TEC than in other TEC subpopulations, with *Notch3* being most highly expressed at E10.5 (Fig. 1A). Of the Notch target genes examined, *Hes1* and *Heyl* showed similar expression patterns to *Notch3* from E12.5. In contrast, and as anticipated, strong expression of the Notch ligand and direct FOXN1 target *Dll4* was initiated at E12.5 (Nowell et al., 2011; Žuklys et al., 2016). At E13.5 and E14.5, *Dll4* was more highly expressed in PLET1^−^ than in PLET1^+^ TECs and was more highly expressed in cTECs than in mTECs, consistent with the *Foxn1* expression pattern and the known expression pattern of *Dll4* in postnatal TECs (Fig. 1A) (Koch et al., 2008). At the protein level, at E13.5 Notch1 was enriched in UEA1^+^ TECs (Notch1^+^ among UEA1^+^, 51.5%±8.4%) compared with UEA1^−^ TECs (24.7%±10.4%) (Fig. 1B). Notch2 and JAG1 were also co-expressed with UEA1 at E14.5, whereas Notch3 was more broadly expressed (Fig. 1C). Furthermore, analysis of the CBF1:H2B-Venus mouse line, which reports Notch signaling (Nowotschin et al., 2013), indicated ongoing or recent NOTCH activity in half of E14.5 UEA1^+^CD205^−^ mTECs compared with only a small minority of cells in the CD205^+^UEA1^−^ ‘cTEC’ population (Fig. 1D). Collectively, these data show that the earliest TECs experience high levels of Notch signaling, while early mTECs remain competent to receive further Notch signals.

### Notch signaling is required for mTEC development

**Fig. 2.**
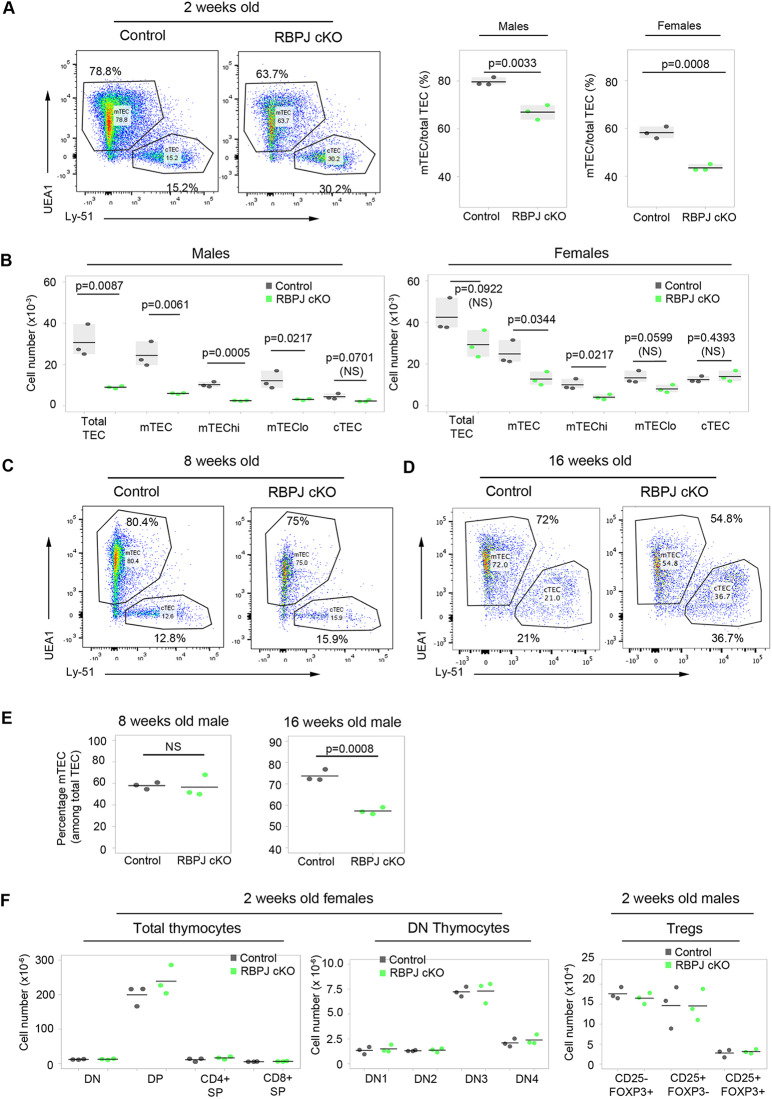
**Loss of *Rbpj* leads to a proportional and numerical reduction of mTECs in postnatal thymus.** (A) Left: representative plots of TEC subset distribution in 2-week-old males. Right: proportion of mTECs among total TECs in 2-week-old males and females. (B) Absolute cell count of total TEC and subpopulations in 2-week-old males (left) and females (right). (C-E) TEC subset distribution in 8- (C,E) and 16- (D,E) week-old males: 78.97±1.56 wild-type 8-week-old mTECs; 78.27±4.98 *Rbpj* cKO 8-week-old mTECs. (F) Left and middle: absolute numbers of thymocyte subsets in 2-week-old females. Right: absolute numbers of CD25^−^FOXP3^−^, CD25^+^FOXP3^−^ and CD25^+^FOXP3^+^ Tregs in 2-week-old males. Tregs were pre-gated as CD4^+^TCRβ^hi^CCR6^−^. (A,B,F) *n*=3 cKO and 3 littermate control mice for male and female. (C-E) 8 weeks, *n*=3 cKO and 3 littermate control male mice; 16 weeks *n*=3 cKO and 3 littermate control male mice from 3 independent litters; results were confirmed in females (not shown). *P* values in pairwise comparisons were calculated using a two-tailed *t*-test.

We next addressed the role of Notch in TEC development, by crossing *Foxn1^Cre^* mice (Gordon et al., 2007) to the *Rbpj^fl/fl^* conditional knockout mouse line (Han et al., 2002). This generated mice in which RBP-Jκ was absent from all TECs and at least some cutaneous epithelial cells, rendering these cells unable to respond to Notch signaling (Han et al., 2002). The recombination efficiency of *Foxn1^Cre^* was close to 100% in E14.5 EPCAM^+^ TECs when tested using a silent GFP (sGFP) reporter (Gilchrist et al., 2003) (Fig. S2), and genotyping indicated complete deletion of *Rbpj* in total TECs purified from 4-week-old *Foxn1^Cre^;RBPJ^fl/fl^* thymi (Fig. S2B). Having validated the *Foxn1^Cre^;RBPJ^fl/fl^* model (herein, *Rbpj* cKO), we next analyzed the effect of TEC-specific loss of RBP-Jκ on the postnatal thymus. This revealed a significant proportional and numerical decrease in mTECs in both male and female *Rbpj* cKO mice at 2 weeks of age (Fig. 2A), with cTEC numbers unaffected (Fig. 2B). The decrease in mTEC numbers reflected reduced numbers of MHC class II^hi^ (mTEC^hi^) and MHC class II^lo^ (mTEC^lo^) TECs in males, and of mTEC^hi^ in females (Fig. 2B). This phenotype normalized by 8 weeks of age, after which a second loss of mTEC was observed (Fig. 2C-E). No other RBP-Jκ-dependent thymic phenotypes were observed: T cell development in the *Rbpj* cKO mice was not blocked at any stage, and no difference in any of the intrathymic Treg precursor or Treg populations (CD25^−^FOXP3^+^, CD25^+^FOXP3^−^, CD25^+^FOXP3^+^) (Lio and Hsieh, 2008; Tai et al., 2013) was detected versus controls (Fig. 2F, Fig. S2D). Thus, the thymic phenotype in the *Rbpj* cKO model appeared TEC specific and affected mTECs but not cTECs. We note that the overall number of TECs was higher in females than in males at 2 weeks of age, in keeping with some previous studies of thymus size, albeit in older mice (Aspinall and Andrew, 2001; Gui et al., 2012), and the proportion of mTECs was higher in males due to increased numbers of cTECs in females compared with males (with no sexual dimorphism in mTEC numbers).

### Notch acts prior to NF-κB signaling to regulate mTEC lineage progression

**Fig. 3.**
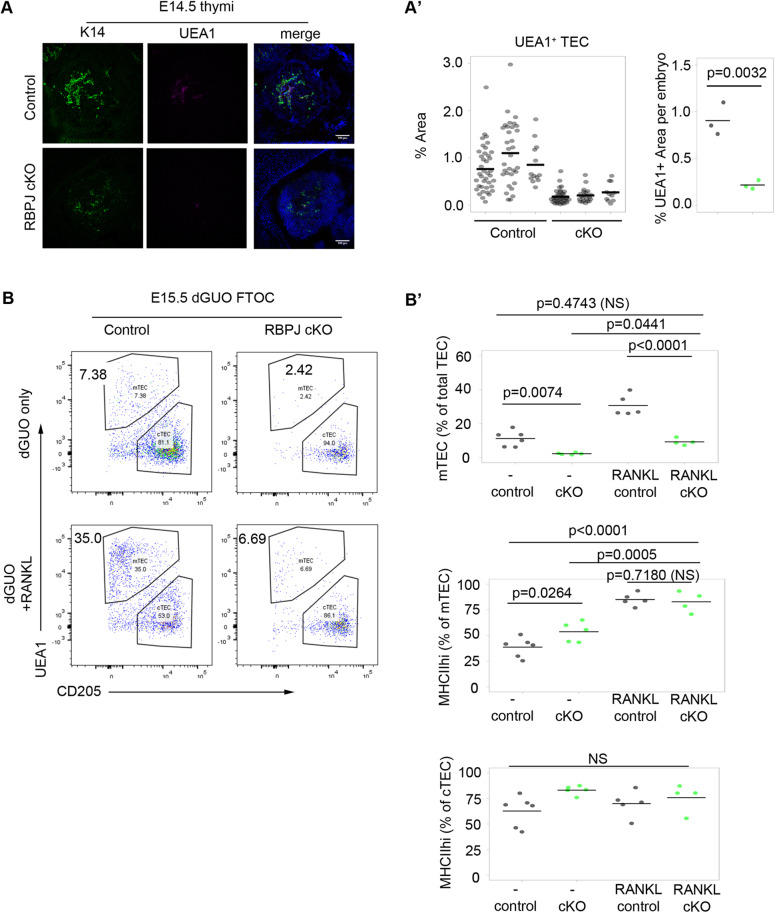
**Notch is required prior to NF-κB signaling in early mTEC development.** (A) Representative transverse sections of embryos of the genotype indicated showing the thymus primordium stained with the mTEC markers K14 and UEA1. DAPI reveals nuclei. (A′) Proportion of pixels in the thymic section (within the outline of DAPI) that stained positive for UEA1. Left plot shows data from each quantified section, grouped by embryo; right plot shows per embryo means from the left plot. (B,B′) E15.5 thymi of the genotypes shown were microdissected and cultured as FTOC for 3 days in dGUO and in the presence of absence of RANKL. (B) Representative plots showing cTEC/mTEC subset distribution after culture. The condition and genotype are as shown. (B′) Quantitation of the percentage of mTECs and the percentage of MHCII^+^ cells in mTEC and cTEC populations. (A,A′) UEA1 images are representative of data collected from 3 cKO and 3 littermate control embryos from 3 separate litters. K14 images are representative of data collected from 4 cKO and 4 control embryos from 4 separate litters. Embryos were snap frozen in OCT. cKO and control embryos were selected for analysis following genotyping. (A′) Left plot: each data point represents a section; right plot, each mean value represents the reconstruction of all thymus-containing sections of an embryo. (B) E15.5 thymi from three litters from a *Foxn1^Cre^;Rbpj^FL/+^*×*Rbpj^FL/FL^* cross were cultured with or without RANKL. Litters were obtained and cultured on different days. Genotypes for each embryo were determined retrospectively. No samples were excluded from the analysis and graphs show all datapoints obtained. For each condition, each *n* represents the thymic lobes from a single embryo; dGuo control, *n*=6; dGuo cKO, *n*=5; RANKL control, *n*=5; RANKL cKO, *n*=4. (A′) *P* values in pairwise comparisons were calculated with a two-tailed *t*-test. (B′) *P* values were calculated using a one-way ANOVA test (two tailed).

To determine whether the *Rbpj* cKO mTEC phenotype arose postnatally or during development, we then analyzed E14.5 control and *Rbpj* cKO thymi using markers characteristic of developing mTECs and cTECs. Fewer K14^+^ and UEA1^+^ presumptive mTECs were present in E14.5 cKO thymi than in littermate controls (Fig. 3A). This indicated that the medullary phenotype was evident by E14.5, 3 days after the onset of Cre expression/*Rbpj* deletion, establishing that Notch signaling is required during emergence of mTEC lineage cells.

The NF-κB pathway ligands RANK ligand (RANKL), lymphotoxin β and CD40L are potent regulators of mTEC development and thymic lympho-epithelial crosstalk (Boehm et al., 2003; Hikosaka et al., 2008). Of these, only RANKL stimulates both proliferation of mTEC and upregulation of the autoimmune regulator (*Aire*). Recent studies have shown that the expression of the RANK receptor and hence responsiveness to RANKL stimulation increases with increasing maturation of mTEC progenitors (Akiyama et al., 2016; Baik et al., 2016; Mouri et al., 2011). To map the requirement for Notch relative to RANK signaling, we turned to the fetal thymic organ culture model (Hare et al., 1999), in an approach similar to that recently used to map the requirements for HDAC3 relative to RANK signaling in mTEC development (Goldfarb et al., 2016). Thus, we cultured E15.5 *Rbpj* cKO and littermate control thymi for 3 days in deoxyguanosine (dGuo)-FTOC conditions (T-cell-depleting FTOC conditions) with or without RANKL. Consistent with the data shown in Figs 2 and 3A, some UEA1^+^ mTEC progenitors arose in the *Foxn1^Cre^Rbpj^fl/fl^* model. Culture of *Rbpj* cKO thymi in RANKL resulted in an approximately threefold proportional increase in mTEC versus unstimulated cKOs and these mTECs displayed a more mature phenotype (MHCII^+^) than controls, indicating that, once generated, these mTEC progenitors respond normally to RANK. Nevertheless, in RANKL-stimulated *Rbpj* cKO thyme, the proportion of mTECs was substantially lower than that in RANKL-stimulated wild-type controls (Fig. 3B,B′), placing the requirement for Notch signaling developmentally upstream of that for RANK. These data establish that Notch signaling acts at an earlier developmental stage than NF-κB signaling to regulate the number of mTEC progenitors and further indicate that, once mTEC progenitors are specified, Notch signaling is dispensable for mTEC differentiation.

### NOTCH signaling is required for specification of the mTEC lineage

The above data would be consistent with Notch regulation of mTEC specification or mTEC progenitor expansion, or both. The *Foxn1^Cre^;Rbpj* cKO model results in deletion of *Rbpj* from around E12.0, with subsequent loss of RBP-Jκ function depending on protein turnover and cell division time. The emergence of mTEC progenitors has, however, been suggested by phenotypic studies to occur independently of FOXN1, possibly at least as early as E10.5 (Hamazaki et al., 2007; Nowell et al., 2011). Thus, the presence of reduced numbers rather than total loss of mTEC progenitors in this model could reflect the relatively late timing of RBP-Jκ deletion, which might allow some mTEC progenitors to emerge prior to loss of Notch signaling-dependent functions in TECs. Therefore, to discriminate between the above models of Notch-mediated regulation of early mTEC development, we determined the effect of blocking Notch signaling in TEC at or prior to mTEC and cTEC lineage divergence. For this, we generated mice in which Notch-mediated transcription is blocked in the developing endoderm before E9.5. We crossed the *Foxa2^T2AiCre^* line with mice carrying the inducible dominant-negative Mastermind allele *Rosa26^loxp-STOP-loxp-dnMAML-IRES-eGFP^* allele (Horn et al., 2012; Maillard et al., 2004) to generate *Foxa2^T2AiCre^;Rosa26^loxp-STOP-loxp-dnMAML-IRES-eGFP^* mice (referred to herein as dnMAML), which exhibit a stronger and much earlier block of NOTCH activity than that in the *Foxn1^Cre^;Rbpj^fl/fl^* (i.e. *Rbpj* cKO) model. dnMAML thymi appeared smaller than controls but contained thymocytes and endothelial networks (Fig. S3).

**Fig. 4.**
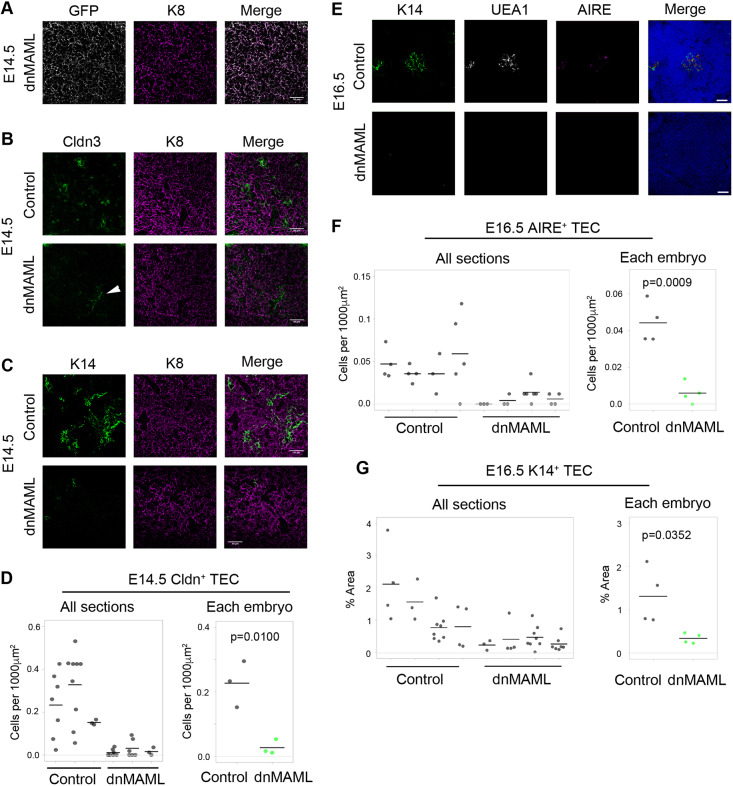
**Notch signaling is an essential mediator of mTEC specification.** (A-C) Representative images of thymi showing (A) the overlap between GFP (recombined cells) and K8 (TECs), and (B,C) staining for mTEC progenitor marker claudin 3 (CLDN3; B), the mTEC marker K14 (C) and epithelial marker K8. Age and genotype are as shown. Scale bars: 50 μm. (D) Quantification of CLDN3^+^ TECs in E14.5 control and dnMAML thymi. Some weakly stained CLDN3^+^ cells colocalized with the endothelial marker CD31 (white arrowhead in B; see also Fig. S7A); hence, for quantification, only CLDN3^+^K8^+^ double-positive cells were counted. (E) Representative images of E16.5 thymi stained for DAPI, UEA1, K14 and AIRE. Scale bars: 50 μm. (F,G) Quantification of AIRE^+^ mTECs as assessed by an unbiased automated counting protocol (F) and of K14^+^ staining (area of marker over the positive threshold/area of thymus defined by DAPI staining) (G) in E16.5 control and dnMAML thymi. *Foxa2^T2iCre^;Rosa26^loxp-STOP-loxp-dnMAML-IRES-eGFP^* and *Foxa2^T2iCre^;Gt(ROSA)26Sor^tm1(EYFP)Cos^* (control) embryos were collected at E14.5 and E16.5. Samples analyzed were littermates. (D,F,G) Each data point represents a section. Mean values from all sections analyzed from the same embryo were used for statistics. E14.5, *n*=3; E16.5, *n*=4 embryos. *P* values were calculated with a two-tailed unpaired *t*-test.

At E14.5, CLDN3^+^ TECs are mTEC-lineage restricted and contain cells with long-term mTEC reconstituting activity (Hamazaki et al., 2007; Sekai et al., 2014). Crucially, at E14.5 this CLDN3^+^ TEC population was completely or almost completely absent from dnMAML thymi (mean reduction of 88% in dnMAML thymi, with some thymi exhibiting a complete loss) (Fig. 4A,B,D; the CLDN3 staining seen in Fig. 4B is restricted to endothelial cells). The number of K14^+^ mTEC was also reduced dramatically in E14.5 dnMAML thymi versus littermate controls (Fig. 4C; the reduction is more pronounced than that in E14.5 *Rbpj* cKO thymi). A profound effect on mTEC development was also evident in E16.5 and E17.5 dnMAML thymi, with some thymi containing no K14^+^, UEA1^+^ or AIRE^+^ mTECs and others containing one or two foci staining for one or more of these markers (Fig. 4E-G; 73% decrease in K14^+^ area; 86% numerical reduction in AIRE^+^ mTECs at E16.5, see also Fig. S4C). These data indicate that blockade of NOTCH-mediated transcription prior to E9.5 results in a near complete block in mTEC progenitor production, effectively resulting in a ‘medulla-less’ thymus.

Thymocyte development was broadly normal in fetal dnMAML thymi at E17.5 (Fig. S4A), consistent with our observations in fetal RBPJ cKO thymi. dnMAML thymi showed a trend towards higher proportions of CD4^−^CD8^−^ double-negative (DN) and CD8^+^ single-positive (SP) thymocytes, and lower proportions of CD4^+^CD8^+^ double-positive (DP) thymocytes, consistent with the changes in Notch ligand expression observed in fetal RBPJ cKO thymi (see below; Table S5, Fig. S8). In addition, preliminary analysis indicated attenuation of positive selection (not shown), and some evidence of perturbed Vγ subset development was observed in dnMAML thymi at E17.5. Vγ subset distribution varied between analysis dates, likely related to the precise developmental time at which the analyses were performed. In one of three litters analyzed, Vγ5 thymocytes were under-represented compared with controls (Fig. S4), consistent with the phenotype observed in perinatal *RANK^−/−^* (*Tnfrsf11a^−/−^*) thymi, which exhibit a marked reduction in Vγ5^hi^ thymocytes (Roberts et al., 2012), and fetal *Aire^−/−^* thymi, in which upregulation of IL7 in *Aire^−/−^* TEC leads to a modest over-representation of Vγ6 thymocytes (Fujikado et al., 2016). In keeping with these data, *Rank* (*Tnfrsf11a*) and *Skint1*, the TEC-expressed selecting determinant required for Vγ5Vδ1 thymocyte development (Turchinovich and Hayday, 2011), were expressed only at very low levels in E14.5 RBPJ cKO TECs, while expression in wild-type controls was as expected (see Fig. 6 below and Table S6). Collectively, these data provide functional corroboration of perturbed mTEC development.

The above conclusion was supported by explant culture of E10.5 3PP. Initial validation of the culture system showed that during 5 days of culture, E10.5 3PP explants undergo morphogenesis, differentiation and self-organization consistent with continuing development of the thymus primordium (Figs S5 and S6A). Culture of E10.5 3PP explants in the presence of the Notch inhibitor DAPT resulted in the specific and near-complete inhibition of mTEC production, evidenced by the absence of UEA1^+^ TECs (Fig. S6B,C). In contrast, the numbers of CD205^+^ cTEC/common TEPCs were not affected (Fig. S6B,C). A few explants contained very rare isolated UEA1^+^ epithelial cells and, strikingly, these rare K14^+^ or UEA1^+^ TECs were exclusively located in the apparent remnant of 3PP lumen (Fig. S6C, arrow), consistent with the localization of CLDN3/4^+^ cells at E10.5 (Hamazaki et al., 2007). Moreover, the number of UEA1^+^ mTECs was unaffected by the presence of RANKL in either control or NOTCH-inhibited conditions (Fig. S6D), indicating that the UEA1^+^ epithelial cells present in the cultures represented early, immature mTECs not yet able to respond to thymic crosstalk (Akiyama et al., 2016; Baik et al., 2016).

Collectively, these data establish an essential role for Notch signaling in the normal emergence of the earliest mTEC progenitors, consistent with an obligatory role in mTEC sublineage specification. They further indicate that, during normal thymus development, mTEC progenitor emergence commences prior to E12.5.

### Notch activity influences TEC progenitor differentiation

Based on the above data, we wished to test whether Notch signaling is permissive or instructive for the specification of mTEC progenitors from the putative common TEPCs. We thus developed a TEC-specific NOTCH gain-of-function model by crossing *Foxn1^Cre^* with *R26-LoxP-stop-LoxP-NICD-IRES-eGFP* (NICD hereafter) mice (Murtaugh et al., 2003) to generate *Foxn1^Cre^;R26-stop-NICD-IRES-eGFP* mice. In this model, high but physiological levels of NICD – and thus constitutively active Notch signaling – are heritably induced in most, if not all, *Foxn1^+^* cells [eGFP expression indicating activation of NICD was seen in over 90% of TECs at E14.5 (Fig. S7; 90.6%±1.3%)].

**Fig. 5.**
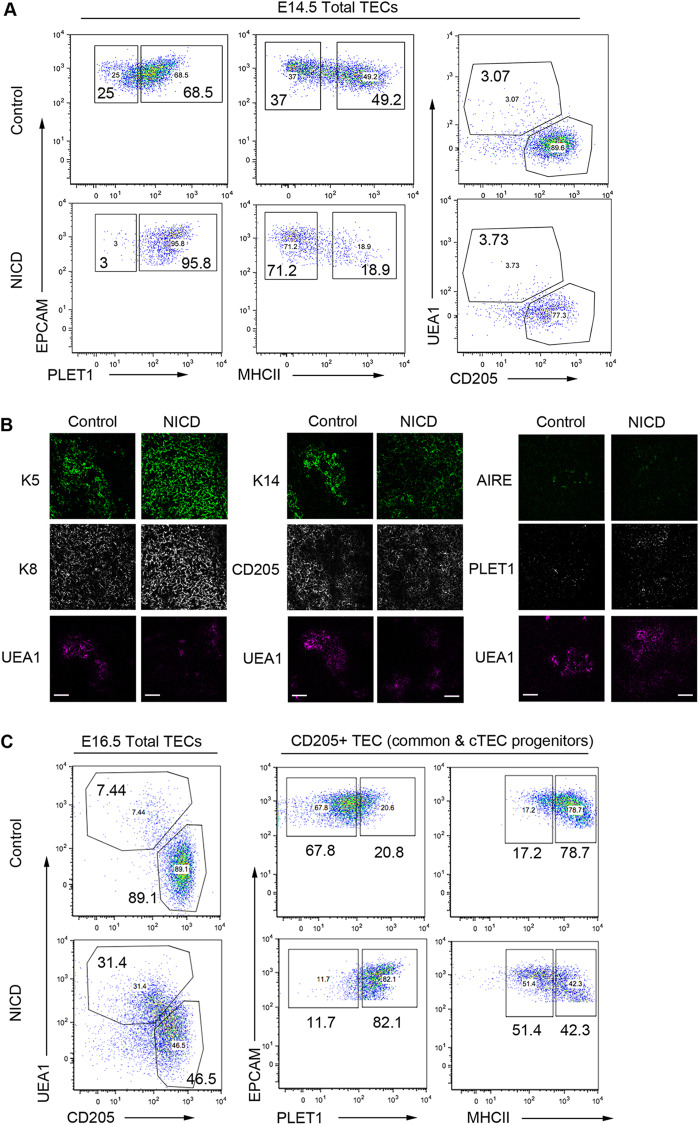
**Outcome of enforced Notch signaling in TEC.** (A) (Left and middle) Representative plots showing E14.5 EpCAM^+^ TEC stained with markers of early progenitor TECs (PLET1), TEC differentiation [MHC class II (MHCII)], mTEC (UEA1) and cTEC (CD205). (Right) Proportions of PLET1^+^MHCII^−^, PLET1^+^MHCII^+^, PLET1^−^MHCII^−^ and PLET1^−^MHCII^+^ TEC in 3 independent E14.5 control and NICD thymi, revealing over-representation of undifferentiated PLET1^+^ TEC and under-representation of differentiated MHCII^+^ TECs in NICD thymi. (B) E16.5 control and NICD thymi stained with the markers shown. Uniform K5^+^ K8^+^ epithelium (left) and expansion of K14 staining into CD205^+^ regions (middle) in NICD compared with clearly demarcated K14^+^ and CD205^+^ zones in controls (right). Both control and NICD thymi express AIRE in UEA1^+^ areas. PLET1 expression is broader in NICD than in controls. Scale bars: 50 μm. (C) Representative plots showing TEC subset distribution in E16.5 thymi after staining for the markers shown. Data after gating on EPCAM^+^ cells (left) and after gating on CD205^+^ cTECs/common TEPCs (right). *Foxn1^Cre^;R26^LSL-NICD-EGFP^* and C57BL/6 control embryos were collected at E14.5 and E16.5. Samples analyzed were from the same litter. E14.5 NICD, *n*=4; E14.5 control, *n*=3; E16.5 NICD, *n*=3; E16.5 control, *n*=3. (B) Images are representative of analysis of thymi from two E16.5 NICD and two control embryos.

To test whether constitutive NICD expression actively promoted mTEC development, we analyzed TEC differentiation at E14.5, assaying progression of TEC differentiation using PLET1 and MHC class II (MHCII) as markers of undifferentiated and differentiated cells, respectively (Nowell et al., 2011). E14.5 NICD thymi exhibited higher proportions of PLET1^+^ and lower proportions of MHCII^+^ TEC than controls, establishing that exposure to continuous Notch signaling from E12.5 onwards resulted in delayed TEC differentiation (Fig. 5A; see also Fig. S1). Analysis of the small population of unrecombined GFP^−^ TECs within the NICD thymus indicated this effect was cell-autonomous, as the expected broad downregulation of PLET1 was observed in these cells (Fig. S7A; see also Fig. S1). The proportion of UEA1^+^ expressing mTECs was unchanged in NICD thymi versus controls, but cells binding high levels of UEA1 were missing (Fig. 5A; NICD, 4.84%±0.21%; control 4.43%±0.34%). Thus, high Notch activity does not drive immediate universal differentiation of mTEC at the expense of cTEC.

Because a rapid expansion of mTEC occurs from E14.5, we also analyzed NICD mice at E16.5. These NICD thymi lacked the clearly demarcated medulla present in age-matched controls (indicated by K5, K14 and UEA1). Compartmental boundaries were indistinct, with a pronounced extension of K5 into K8^hi^ CD205^+^ regions and more extensive PLET1^+^ areas, suggesting that most TEC had a progenitor cell phenotype (Fig. 5B) (Bennett et al., 2002; Gill et al., 2002; Klug et al., 1998). The NICD sections exhibited similar proportions of AIRE^+^ mTECs to control thymi. Flow cytometry analysis also showed that the UEA1^+^ and CD205^+^ populations were less clearly defined, with many cells exhibiting an apparently intermediate phenotype (Fig. 5C). Thus, at E16.5 the NICD thymi contained fivefold more UEA1^+^ mTECs (35.7%±7.6%) than control thymi (6.6%±1.1%), but the proportion of UEA1^+^ TECs expressing the highest levels of UEA1 was diminished (Fig. 5C). Additionally, the CD205^+^ cTEC/common progenitors displayed considerably higher PLET1 and lower MHCII levels than controls, consistent with a continued delay/block in cTEC differentiation (Fig. 5C).

Collectively, these data establish that overexpression of Notch promotes, but does not dictate, mTEC emergence from the common TEPC and additionally blocks or substantially delays cTEC lineage progression.

### Impact of Notch signaling modulation on gene expression in fetal TECs

To further interrogate the phenotype of NOTCH loss- and gain-of-function models, we analyzed the transcriptome of fetal TECs, aiming to identify mechanisms regulated by Notch signaling within specific TEC populations. For both *Rbpj* cKO and control thymi, we performed RNAseq analysis on E12.5 PLET1^+^ TEPCs and E14.5 PLET1^+^ and PLET1^−^ TECs, while for NICD at E14.5 we analyzed only PLET1^+^ TEC, as most NICD TEC were PLET1^+^ at this timepoint (Fig. 5A; deposited in GEO under accession number GSE100314. A trend suggestive of downregulation of some Notch family and Notch target genes was indicated in RNAseq analysis of E14.5 PLET1^+^ *Rbpj* cKO versus control TEC (Table S5, Fig. S8) and confirmed by RT-qPCR (Fig. S9), pointing to a positive-feedback loop regulating Notch-signaling competence. Conversely, several Notch family genes were significantly upregulated in E14.5 NICD TEC versus controls (Table S5, Fig. S8).

**Fig. 6.**
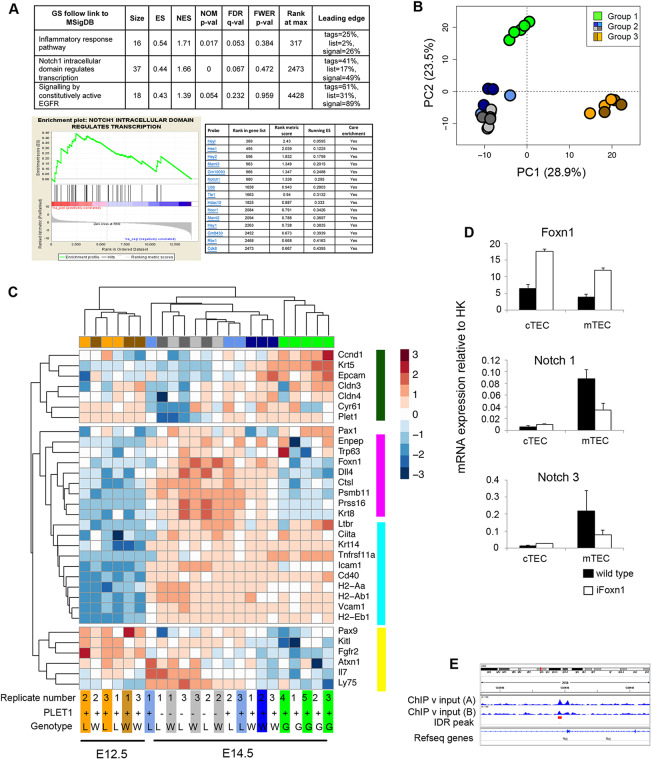
**Transcriptome analysis of Notch loss- and gain-of-function mutants.** (A) Pathway analysis of the E14.5 NICD and E14.5 controls identified three signaling pathways as enriched (FDR≤0.25) in E14.5 NICD versus E14.5 control thymi (top). GSEA enrichment plot for the Notch signaling pathway (bottom left). Leading edge subset genes contributing to the enrichment for Notch signaling pathway (bottom right). (B) PCA of *Rbpj* cKO, wild-type and NICD TECs at the ages shown (500 most variable genes). Group 1, E14.5 NICD samples; group 2, E14.5 PLET1^+^ and PLET1^−^ *Rbpj* cKO and controls; and group 3, E12.5 *Rbpj* cKO and controls. (C) Heatmap of lineage-specific genes among all groups of samples shown in the PCA above. Colors at the top and bottom of the heatmap indicate clustering of samples per group, while side colors indicate groups of genes regulated similarly across all conditions. Groups: E12.5 wild type, brown; E12.5 *Rbpj* cKO, orange; E14.5 wild-type PLET1^+^, dark blue; E14.5 wild-type PLET1^−^, light gray; E14.5 *Rbpj* cKO PLET1^+^, light blue; E14.5 *Rbpj* cKO PLET1^−^, dark gray; W, wild type; L, loss of function (*Rbpj* cKO); G, gain of function (NICD). (D) RT-qPCR analysis of sorted cTECs and mTECs from E17.5 wild-type and iFoxn1 thymi for the genes shown. Data are mean±s.d. (E) Genomic locus of *Rbpj* showing Foxn1 peaks identified by Zuklys et al. (2016). (A-C) To obtain the E12.5 and E14.5 cKO and wild-type samples, thymi were microdissected from E12.5 and E14.5 embryos generated from a *Foxn1^Cre^;Rbpj^FL/+^*×*Rbpj^FL/FL^* cross and TECs were obtained by flow cytometric cell sorting. Following genotyping, cells from three cKO and three control samples were processed for sequencing. The E12.5 and E14.5 samples were each obtained from two separate litters, on two separate days for each timepoint. To obtain the E14.5 NICD samples, thymi were microdissected from five E14.5 Foxn1Cre; R26^LSL-NICD-EGFP^ embryos of the same litter, TECs were obtained by flow cytometric cell sorting and the samples processed for sequencing. (D) *n*=3, where each *n* represents TECs sorted from pooled embryos from a single litter of E17.5 iFoxn1 or wild-type embryos.

Independent signaling pathway enrichment analysis using all genes differentially expressed between the E14.5 NICD and wild-type datasets also revealed the Notch pathway as one of those most affected by NICD overexpression (Fig. 6A). In addition, we found significant upregulation of the EGFR pathway, which is known to promote the proliferation of mTEC precursors (Satoh et al., 2016), and of several collagen genes (annotated as ‘Inflammatory Response Pathway’), suggesting that Notch signaling may play a role in endowing proliferative capacity on nascent mTECs and in regulating TEPC differentiation by modifying extracellular matrix (Baghdadi et al., 2018). Neither *Foxn1* nor *Plet1* expression was significantly affected by loss of *Rbpj* (Tables S5 and S6, Figs S8 and S9). The bHLH transcription factor *Ascl1* was downregulated in *Rbpj* cKO TECs, and was also highly enriched in mTECs in wild-type mice, with strong upregulation occurring co-temporally with medullary expansion at E14.5 (Figs S8, S9 and S10A). This suggested that ASCL1 might act downstream of Notch in mTEC lineage regulation. However, no differences in thymic size, organization or cellularity were detected in *Ascl1^−/−^* thymi (Guillemot et al., 1993) at E17.5 (Fig. S10B), apparently ruling out this hypothesis.

Principal component analysis (PCA) clustered the E12.5 and E14.5 PLET1^+^ *Rbpj* cKO, and wild-type and E14.5 PLET1^+^ NICD datasets into three groups: E14.5 NICD samples (group 1); E14.5 PLET1^+^ and PLET1^−^ *Rbpj* cKO and controls (group 2; see also Fig. S11); and E12.5 *Rbpj* cKO and controls (group 3) (Fig. 6B). The broad PCA analysis (Fig. 6B) separated the samples by developmental stage (PC1) and PLET1 level (PC2; PC2 is not solely PLET1), with group 1 positioned between group 2 and group 3 in PC1. Overall, the PCA is consistent with E14.5 NICD TECs exhibiting at least a partial developmental delay (in keeping with conclusions from Fig. 5) or with sustained NICD expression in early TECs inducing a distinct cell state that is not found or is very rare in the early wild-type fetal thymus.

Consistent with these possibilities, clustering analysis revealed differential effects of Notch signaling perturbation on markers associated with differentiation into the cTEC and mTEC sub-lineages, general TEC maturation or the earliest TEPC state. In particular, genes associated with cTEC lineage identity (*Ctsl*, *Dll4*, *Psmb11*, *Prss16*, *Krt8* and *Ly75*) were upregulated normally from E12.5 to E14.5 in the *Rbpj* cKO samples but were expressed at levels similar to E12.5 wild type in the E14.5 NICD samples (Fig. 6C), consistent with maintained Notch signaling imposing a block on cTEC generation from the common TEPC/early cTEC progenitor. *Foxn1* also exhibited this expression pattern (Fig. 6C), and indeed many genes in this panel are direct FOXN1 targets (Calderón and Boehm, 2012; Nowell et al., 2011; Žuklys et al., 2016). Notably, constitutive overexpression of FOXN1 in fetal TEC led to downregulation of a number of Notch family and Notch target genes (Fig. 6D; data not shown), suggesting that induction of FOXN1 may downregulate Notch signaling in TECs during normal development *in vivo*. Consistent with this, our re-analysis of published FOXN1 ChIP-seq data (Žuklys et al., 2016) indicated *Rbpj* as a direct FOXN1 target (Fig. 6E). Moreover, Žuklys and colleagues (Žuklys et al., 2016) identified several known Notch targets and modulators as FOXN1 targets (*Heyl*, *Hes6*, *Deltex4* and *Fbxw7*). The relative downregulation of *Foxn1* resulting from sustained NICD expression in early fetal TECs (Fig. 6C, Fig. S8) thus suggests the possibility of reciprocal inhibition.

Other genes associated with both cTEC and mTEC differentiation were unaffected or only marginally affected by the Notch signaling gain- or loss-of-function mutations (Fig. 6C and Table S6). In contrast, markers associated with the mTEC sub-lineage (*Krt5* and *Epcam*) were strongly upregulated in the E14.5 NICD samples compared with controls, and these genes also clustered with other genes normally strongly downregulated from E12.5 to E14.5 (*Cldn3*, *Cldn4*, *Cyr61*, *Plet1* and *Ccnd1*). *Tnfrs11a*, the gene encoding RANK, was also significantly upregulated in the E14.5 NICD samples (Fig. 6C and Table S6), and was expressed at much lower levels in E14.5 Rbpj cKO than controls. Finally, a category including *Pax9*, *KitL* and *Fgfr2*, which are normally highly expressed at E12.5, was markedly downregulated in the E14.5 NICD compared with other E14.5 samples (Fig. 6C).

**Fig. 7.**
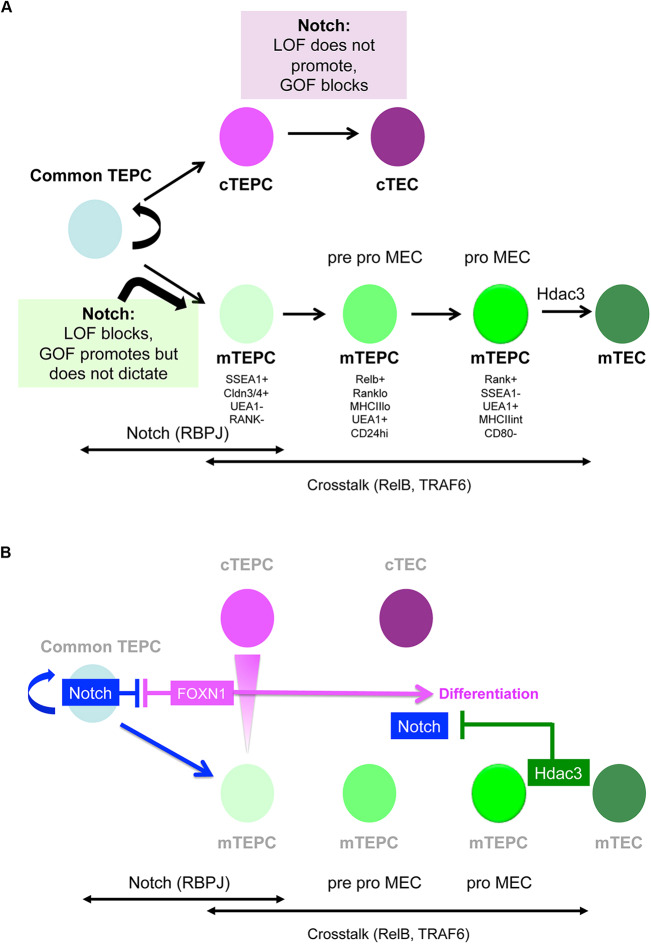
**Model for Notch signaling regulation of early TEC development.** Schematic diagrams presenting the model of early TEC development supported by the findings presented herein. (A) Notch signalling has an essential role in the differentiation of early fetal TECs: its loss of function results in mTEC hypoplasia, while its gain of function leads to TEPC maturation arrest. Notch activity precedes crosstalk-dependent further expansion and maturation of mTECs. (B) The Notch pathway in the context of a broader regulatory network. In early TEC differentiation, Notch influences and may be influenced by FOXN1, whereas it is suppressed by HDAC3 in postnatal mTECs.

Overall, we conclude that upregulation of Notch signaling in TECs during early thymus development at least partially blocks cTEC differentiation and promotes, but does not dictate, mTEC development, suggesting that Notch regulates not only mTEC specification but also maintenance of the fetal common TEPCs (Fig. 7).

## DISCUSSION

We have used conditional loss- and gain-of-function approaches together with pharmacological inhibition to investigate the role of Notch signaling in TEC. Our data show, based on TEC-specific RBP-Jκ deletion, γ-secretase inhibition in FTOC and enforced dnMAML expression in the developing endoderm from E9.5, that Notch activity is essential for mTEC development. Specifically, they establish that Notch signaling is required for the emergence of the mTEC sub-lineage from the putative bipotent TEC progenitor, strongly suggesting that Notch regulates mTEC specification. Additionally, they demonstrate that Notch signaling, although essential, is permissive rather than instructive for mTEC development, and indicate a further role for Notch in regulating exit from the early bipotent TEPC state into mTEC and cTEC differentiation. These findings, summarized schematically in Fig. 7, raise several issues that are discussed below.

### Timing of the Notch signaling requirement

Notch signaling has been shown to regulate distinct events in the different developmental stages of a tissue (Hartman et al., 2010; Radtke et al., 2004; Shih et al., 2012). A recent study reported that Notch activity is enriched in cTECs and that repression of Notch by HDAC3 is important for expansion/maintenance of developing mTECs (Goldfarb et al., 2016). This study analyzed the same Notch overexpression line used herein, but at the later time-points of 10 days and 6 weeks postnatally (Goldfarb et al., 2016). The conclusions of this and our own studies are entirely compatible, with the data presented here establishing a requirement for Notch signaling at the earliest stages of TEC lineage divergence, and the data of Goldfarb indicating that downregulation of Notch signaling is required for later stages of mTEC differentiation (Goldfarb et al., 2016). It is also possible that Notch has secondary roles in TECs subsequent to its initial role in mTEC specification.

### Thymic crosstalk

The NF-κB pathway plays a vital role in mTEC development and consequently in the establishment of central tolerance (Akiyama et al., 2005; Burkly et al., 1995; Kajiura et al., 2004). Recent studies using transcriptomic and functional assays have increased clarity on how the NF-κB ligands through which thymic crosstalk occurs regulate mTEC maturation (Akiyama et al., 2016; Bichele et al., 2016; Desanti et al., 2012; Mouri et al., 2011). In particular, Akiyama and colleagues identified two separable UEA1^+^ mTEC progenitor stages, pro-pMECs and pMECs, based on the expression of RANK, MHCII and CD24 (Akiyama et al., 2016). The transition from the more primitive pro-pMECs to pMECs depends on RELB, whereas further maturation from pMECs is TRAF6 dependent. Crucially, both pro-pMECs and pMECs respond to induction by RANKL in T cell-depleted FTOC (Akiyama et al., 2016). Our exploratory experiments suggested that Notch inhibition attenuated RANKL stimulation in E15.5 FTOC (data not shown), which we initially interpreted to indicate potential synergy between Notch and NF-κB. However, analysis of E15.5 *Rbpj* cKO versus wild-type FTOC indicated that NF-κB activation of already specified mTEC progenitors was unaffected by lack of Notch signaling responsiveness: although the block in mTEC development was more severe in the *Rbpj* cKO FTOC, the few mTECs that were present could be stimulated by RANK, indicating the presence of pMECs and/or pro-pMECs. The attenuation of RANKL stimulation upon DAPT treatment of E15.5 wild-type FTOC thus suggests that mTEC specification is still ongoing at E15.5. However, we also observed that mTEC clusters in *Rbpj* cKO thymi tended to be smaller than those in controls, and therefore the possibility that, in addition to regulating mTEC specification, Notch also regulates the initial expansion of mTEC progenitors cannot be ruled out. Indeed, our data reveal EGFR signaling as a major target of Notch during early TEC development.

In contrast, our data show that, although E10.5 3PP explants can generate UEA1^+^ mTECs and CD205^+^ cTEC/progenitors in culture, these UEA1^+^ mTECs do not respond to RANKL. It is thus likely that the UEA1^+^ cells in these explants represent an even more primitive mTEC progenitor state than pro-pMECs. Of note, some DAPT-treated E10.5 3PP explants produced no UEA1^+^ mTECs, and thus mTEC specification can be completely suppressed in the absence of Notch signaling. Taken together, these results suggest that, although Notch and NF-κB are both required for mTEC development, the two pathways act sequentially but independently.

### Notch regulation of mTEC progenitor emergence

The loss of mTECs in Notch loss-of-function models could be explained by three hypotheses: (1) Notch might regulate the decision of bipotent TEPCs to become mTECs (In this model, in the absence of Notch signaling, bipotent progenitors would fail to commit to mTEC fate and over time become cTECs instead.); (2) alternatively, high levels of Notch signaling might dictate that TEPCs remain bipotent, with cells that experience lower Notch committing to the cTEC lineage (Unlike the ‘specification hypothesis’, in this scenario mTECs would fail to emerge in the absence of Notch signaling because the bipotent TEPCs undergo premature differentiation into cTECs, exhausting the pool that retains the potential for mTEC generation.); and (3) finally, Notch might be required for the proliferation of specialized mTEC progenitors (In this case we would expect the perturbation to affect only mTECs and not cTECs or bipotent progenitors.).

We conclude from the gain-of-function data that enhanced Notch activity neither switches all TECs to become mTECs, nor affects only mTECs. Instead, Notch activity is necessary but not sufficient for mTEC fate in the developmental timeframe investigated. Despite the caveats with established markers, the considerable shift towards a PLET1^+^MHCII^−^ (Fig. 5A,C) K5^+^ K8^+^ (Fig. 5B) phenotype suggests a more immature, TEPC-like state as the primary phenotype resulting from high Notch activity. Indeed, the transcriptome of E14.5 NICD TECs occupies a state that is separate from both E12.5 TEPCs and age-matched controls, while sharing certain features with both clusters. As development progresses from E14.5 to E16.5, many TECs do upregulate the mTEC markers UEA1 and K14, indicating that high Notch activity is compatible with acquisition of mTEC fate. Importantly, the NICD^+^ UEA1^+^ mTECs at E16.5 display comparable maturation status with controls, whereas CD205^+^ cTEC/common TEPCs continue to exhibit a primitive phenotype (Fig. 5). These data suggest that once mTECs are specified, further development is independent of Notch signaling.

The gain-of-function results also support our hypothesis that Notch operates at the TEC progenitor level, while opposing the model that Notch activity only influences mTECs. However, it does not rule out the specification model. Although retention of an early progenitor state seems to be the primary outcome of enforced Notch signaling, the proportion of mTEC in the E16.5 gain-of-function thymi is higher than controls. Several factors may be in play in this second phase. The duration of signaling has been shown to result in the temporal adaptation of sensitivity in several pathways (reviewed by Kutejova et al., 2009). Moreover, instead of a simple ON/OFF response, the Notch response may be graded, as in the case of inner ear (Petrovic et al., 2014) and pancreas development (Shih et al., 2012). mTEC specification may require higher levels of Notch, which could, for example, be achieved by positive feedback above the level of that imposed by the enforced NICD expression in the NICD hemizygous mice used in these experiments. Variables independent from Notch may also play a part. A potential candidate is FOXN1, which drives TEPCs out of the primitive undifferentiated state and into differentiation (Nowell et al., 2011); indeed our data indicate interplay between FOXN1 expression levels and Notch activity (as depicted in Fig. 7). In addition to the direct cross-regulation suggested from our analysis, FOXN1-mediated repression of Notch activity could be reinforced via its direct targets DLL4 and FBXW7; the former may mediate cis-inhibition of NOTCH receptors, while the latter has been shown to enhance the degradation of NICD (Carrieri and Dale, 2016; del Álamo et al., 2011). We note that the thymic phenotype of the Notch gain-of-function mutant reported here resembles those of *Foxn1^R/−^* (Nowell et al., 2011) and *Foxn1^Cre^;iTbx1* (Reeh et al., 2014) mutant mice, in which exit from the earliest TEPC compartment is also severely perturbed owing to the inability to express normal levels of FOXN1.

One of the long-term goals of the field is to create fully functional thymus organoids from TECs derived from pluripotent stem cells or by direct conversion from unrelated cell types (reviewed by Bredenkamp et al., 2015). Understanding the duration of TEPC bipotency, lineage plasticity and Notch activity would improve protocols and inform strategies in this regard. Our data predict that, by manipulating the levels of Notch signaling TEPCs experience, it may be possible to produce more homogenous populations of TEC subsets, including TEPC. However, the complexities indicated from studies on Notch in other organs, together with the potential for differential effects on TEC at different stages of lineage progression, suggest that further advances in this direction will require caution and precision.

## MATERIALS AND METHODS

### Mice

CBAxC57BL/6 F1 mice were used for isolation of fetal TEC. For timed matings, C57BL/6 females were housed with CBA males, and noon of the day of the vaginal plug was taken as E0.5. Representative data shown were obtained from littermates or, when not possible, embryos sharing the same plug date. *Foxn1^Cre^* (Gordon et al., 2007), *Rbpj* conditional knockout (Han et al., 2002), *Rosa26-stop-NICD* (Murtaugh et al., 2003), *CBF1-Venus* (Nowotschin et al., 2013), *Ascl1^−/−^* (Guillemot et al., 1993), *Rosa26^CreERt2/CAG-Foxn1-IRES-GFP^* (iFoxn1) (Bredenkamp et al., 2014), and *Foxa2^T2iCre^;Rosa26^loxp-STOP-loxp-dnMAML-IRES-eGFP^* and *Foxa2^T2iCre^;Gt(ROSA)26So^rtm1(EYFP)Cos^* (Horn et al., 2012; Maillard et al., 2004) mice were as described. All animals were housed and bred at the CRM animal facilities, except for the *Ascl1^−/−^* strain, which was housed and bred at the NIMR (Mill Hill, London), the *Rosa26NICD* strain (Murtaugh et al., 2003), which was housed and bred at EPFL (Lausanne, Switzerland), and the *Foxa2^T2iCre^;Rosa26^loxp-STOP-loxp-dnMAML-IRES-eGFP^* (Horn et al., 2012; Maillard et al., 2004) and *Gt(ROSA)26Sor^tm1(EYFP)Cos^* (R26LSL-YFP) (Srinivas et al., 2001) strains, which were housed and bred at DanStem (University of Copenhagen, Denmark). *Foxn1^Cre^* (Gordon et al., 2007) were also housed and bred at EPFL. All experimental procedures were conducted in compliance with the UK Home Office Animals (Scientific Procedures) Act 1986. Primers used for genotyping are provided in Table S4.

### Thymus dissociation

Postnatal thymi were dissociated in 1.25 mg/ml collagenase D (Roche) and subsequently in 1.25 mg/ml collagenase/dispase (Roche) diluted in RPMI medium (Life Technologies). DNaseI (Lorne; 0.05 mg/ml) was added to the buffer to minimize cell adhesion. Fetal thymi were dissociated for 20 min using a PBS-based buffer consisting of 1.25 mg/ml collagenase D, 1.4 mg/ml hyaluronidase (Sigma) and 0.05 mg/ml DNaseI. After digestion, cells were spun down and digested in 1× trypsin for 2 min. Cell suspension was then filtered through 70 μm cell strainer (Corning) to remove clumps.

### Flow cytometry

Adult thymi and RFTOC were processed for flow cytometric sorting and analysis as previously described (Bredenkamp et al., 2014; Nowell et al., 2011). For analysis and sorting, adult thymic tissue was depleted of T cells using anti-CD45 MACS beads (Miltenyi Biotec); fetal tissue was not T-cell depleted. Cell counts were carried out using a BioRad cell counter and slides, where required. Sorting and analysis were performed using a BD FACS Aria II and a BD LSR Fortessa, respectively, at the CRM (University of Edinburgh). For *Rosa26NICD* TECs, sorting was performed on a BD FACS Aria II at the University of Lausanne (Epalinges). Sorting protocols were identical for all cell-isolation experiments. All flow cytometry data were analyzed using FlowJo Version 9.7.6 (Tree Star).

### Immunohistochemistry

Immunohistochemistry was performed as described previously (Gordon et al., 2004). Appropriate isotype and negative controls were included in all experiments. For detection of immunofluorescence, slides were examined with Leica SP2, SPE and SP8 confocal microscopes. Images presented are of single optical sections. Fiji software (Schindelin et al., 2012) was used to quantify the surface area of positive staining and the thymic section. Volume percentage of K14^+^ or UEA1^+^ regions in an embryo was defined as total area of positive staining divided by total area of thymic section.

For the quantification of AIRE^+^ TECs shown in Fig. 4, we used an automated counting method to remove bias from the analysis. In brief, we set an automatic threshold for the AIRE images using the Rényi's entropy setting in Fiji, such that pixels that were brighter than the average (i.e. background) were scored as positive. This was used to create a black and white image from the input AIRE staining that was then merged with the K14 co-stains. The criterion used to identify an AIRE^+^ TEC was a cluster of white pixels (representing AIRE) surrounded by a K14^+^ circle (representing K14^+^ cytoplasm).

### Antibodies

The antibodies used for immunohistochemistry and flow cytometry were as listed in Tables S1 and S2.

### Fetal thymus organ culture

E15.5 FTOCs were maintained on a Millipore membrane raft floating on DMEM supplemented with 10% FCS and L-glutamine. E10.5 third pharyngeal pouches were submerged and allowed to settle on thin matrigel (Corning), then cultured in N2B27 (DMEM) medium, 20 ng/ml BMP4 (Peprotech), 20 ng/ml FGF8 (Peprotech), penicillin/streptomycin and 1 μg/ml heparin. Where DAPT (Tocris) or deoxyguanosine (dGUO; Sigma) were used, the equivalent amount of DMSO was added to the control medium. RANKL (Peprotech) was used at 500 ng/ml.

### Quantitative real-time PCR

RT-qPCR was performed as previously described (Bredenkamp et al., 2014) on 50-200 cells per sample. Data are shown after normalization to the geometric mean of three control genes (*Hprt*, *Ywhaz* and *Hmbs*). Data analysis was carried out using LightCycler 1.5 software and the ΔCt method (Livak and Schmittgen, 2001). Primers used for RT-qPCR are as shown in Table S3.

### RNA-seq

100 cells were sorted directly into Smartseq2 lysis buffer (Picelli et al., 2013) at the CRM (University of Edinburgh) (*Rbpj* cKO and littermate control samples) or at the University of Lausanne (Epalinges) (*Rosa26NICD* samples). Sorted samples were immediately frozen on dry ice and were then shipped to the WIMM (University of Oxford) for library preparation. The libraries were then prepared and sequenced at the Wellcome Trust Centre for Human Genetics, University of Oxford. Quality control (QC) of the raw reads by FastQC (Andrews, 2010) indicated small amount of adaptor contamination and few low-quality reads; therefore, the raw data were trimmed with Trimmomatic (Bolger et al., 2014) using default parameters for PE reads and the cropping option specific to the Nextera PE adapters. Only paired reads that passed QC were aligned with STAR against the mouse genome assembly (GRCm28 – Ensembl 87) and the aligned reads were assigned to genes with featureCounts (Liao et al., 2014). The resulting count tables were imported to R for further normalization and analysis. Batch effect correction was applied for the within group lane effects; however, some batch effects could not be corrected. This applied to the potential for a laboratory effect between the E14.5 NICD and all other samples, as the E14.5 NICD sample was collected at EPFL Lausanne. However, the same thymus dissociation and cell-sorting protocols, and the same make and model of cell sorter were used, and the subsequent sample processing was performed at the University of Oxford using the same protocol as for all the other samples. To control for this, the expression levels of housekeeping genes were determined for all samples and were not biased in any particular groups (Fig. S11B).

Differential expression analysis was performed using the LIMMA package and voom (Ritchie et al., 2015) from Bioconductor (Gentleman et al., 2004), and a threshold of FDR≤0.05 was set to define genes that change with significance between the different datasets. The table of all differentially expressed genes and their fold changes was used as a pre-ranked list in GSEA (Subramanian et al., 2005) against the ConsensusPathDB (Kamburov et al., 2011) to predict signaling pathways that are enriched between the wild-type and NICD samples. Pathways were defined as enriched if they had a FDR value of less than or equal to 0.25 (default significance criteria for GSEA).

### ChIP-seq

Publicly available data deposited GEO accession number GSE75219 (Žuklys et al., 2016) were reanalyzed as follows. QC of the raw reads by FastQC (Andrews, 2010) indicated a few low-quality reads; these were therefore removed trimming the raw data with Trimmomatic (Bolger et al., 2014) using default parameters for PE reads. Read mapping was performed with Bowtie2 (Langmead and Salzberg, 2012) with default parameters; MACS2 (Zhang et al., 2008) was used with a lenient *P*-value threshold of 1×10^−3^ to call peaks. The IDR pipeline (Li et al., 2011) was followed to call confident peaks among replicates (IDR≤0.05).

### Statistics

Statistical analysis was performed using the GraphPad Prism 7.02 software. Student's *t*-test (two-tailed, unpaired) was performed for pair-wise comparisons. Multiple comparison procedures were performed with one-way ANOVA test (two tailed), as appropriate for normally distributed data (normal distribution was tested using χ^2^ goodness of fit). The alpha level is taken as 0.05. Errors where shown are standard deviations (s.d.). Sample sizes of at least *n*=3 were used for all analyses, except where indicated. Where plotted, averages shown are means. For all analyses, *n* represents the number of independent biological experiments. No statistical method was used to predetermine sample size, the experiments were not randomized and the investigators were not blinded to allocation during experiments and outcome assessment. There were no limitations to repeatability of the experiments. No samples were excluded from the analysis, except for a small number of extreme outliers related to the flow cytometric analysis of fetal dnMAML thymi; these omissions are noted in the relevant figure legends. Graphs were prepared using the PlotsofData App (Postma and Goedhart, 2019) and R package ggplot (Wickham, 2016).

## Supplementary Material

Supplementary information
