# Supplementary material for: Canonical Notch signaling controls the early thymic epithelial progenitor cell state and emergence of the medullary epithelial lineage in fetal thymus development
Source: Development. 2020 Jun 22;147(12):dev178582. doi: 10.1242/dev.178582 (PMC7328009; doi:10.1242/dev.178582)
Supplement: Supplementary information [file develop-147-178582-s1.pdf]

Table S1: Antibodies used for flow cytometry

Antigen	Conjugate	Clone	Supplier	Dilution
EPCAM	APC	G8.8	eBioscience	1:200
EPCAM	PE	G8.8	Biolegend	1:800
EPCAM	APCCy7	G8.8	Biolegend	1:200
MHCII	PE	M5/114.15.2	BD Pharmingen	1:200
MHCII	APCeFluor780	M5/114.15.2	eBioscience	1:200
MHCII	PECy7	M5/114.15.2	eBioscience	1:200
CD45	APC	30-F11	eBioscience	1:1000
CD45	APCeFluor780	30-F11	eBioscience	1:1000
CD45	PerCP Cy5.5	30-F11	eBioscience	1:200
NOTCH1	APC	22E5	eBioscience	1:100
UEA1	Biotin	#B-1065	Vector	1:500
CD205	PECy7	205yetka	eBioscience	1:20
LY51	PE	6C3	eBioscience	1:200
CD4	BV650	RM4-5	Biolegend	1:800
CD4	PECy7	RM4-5	eBioscience	1:1600
CD8a	PerCP Cy5.5	53-6.7	eBioscience	1:200
CD25	PE	PC61.5	eBioscience	1:1600
CD44	APC	IM7	Biolegend	1:1600
CD11b	FITC	M1/70	eBioscience	1:1000
CD11c	PerCP Cy5.5	N418	Biolegend	1:200
GR1	FITC	RB6-8C5	Biolegend	1:1000
NK1.1	FITC	PK136	Biolegend	1:1000
CD62L	BV605	MEL-14	Biolegend	1:400
CD69	BV421	H1.2F3	Biolegend	1:100
MHC Class I	BV510	AF6-88.5	Biolegend	1:400
CD31	PerCP Cy5.5	390	Biolegend	1:200
TER119	PerCP Cy5.5	Ter119	eBioscience	1:200
B220	FITC	RA3-6B2	eBioscience	1:1000
TCRβ	PerCP Cy5.5	H57-597	Biolegend	1:200
CCR6	PECy7	G034E3	Biolegend	1:20

FOXP3	PE	FJK-16S	eBioscience	1:100
Pan TCR	Pe/Cy7	GL3	Biolegend	1:400
Vγ1	PerCp/Cy5.5	2.11	Biolegend	1:200
Vγ4	APC	UC3-10A6	Biolegend	1:400
Vγ5	BV605	536	BD Biosciences	1:50
Vγ1 + Vγ2	Anti-rat – PE	4B2.9	Biolegend	1:200
PLET1	None	1D4	Homemade	Undiluted
Streptavidin	BV650	#405231	Biolegend	1:500
TCRγ/δ	APC	eBioGL3	eBioscience	1:100
Ki67	PE	SolA15	eBioscience	1:400
Activated Caspase 3	PE	C92-605	BD Pharmingen	1:10

Table S2: Antibodies used for immunohistochemistry

Antigen	Host species	Clone	Supplier	Dilution
PanK	Rabbit	Z0622	DAKO	1:250
K5	Rabbit	AF138	BD	1:250
K8	Rat	Troma1	DSHB	1:250
K14	Rabbit	AF64	Covance	1:800
CLDN3	Rabbit	AB15102	Abcam	1:10
NOTCH2	Rabbit	25-255	Santa Cruz	1:50
NOTCH3	Rabbit	AB23426	Abcam	1:200
JAG1	Rabbit	H114	Santa Cruz	1:50
AIRE	Rabbit	M300	Santa Cruz	1:200
PLET1	Rat	1D4	Homemade	1:10
CD205	Rat	NLDC-145	AbD Serotec	1:10
UEA1	Biotin	#L-1060	Vector	1:500
AIRE	Rat	5H12	eBioscience	1:100
Rat IgG isotype	Rat	RTK4530	BioLegend	1:1000
Rabbit IgG isotype	Rabbit	#550875	BD	1:1000

Table S3. Primers used for RT-qPCR

Gene name	Primer sequence (5'- 3')
Notch1 F	GGATGCTGACTGCATGGAT
	AATCATGAGGGGTGTGAAGC
Notch2	TGCCTGTTTGACAACTTTGAGT
	GTGGTCTGCACAGTATTTGTCAT
Notch3	AGCTGGGTCCTGAGGTGAT
	AGACAGAGCCGGTTGTCAAT
Jag1	GAGGCGTCCTCTGAAAAACA
	ACCCAAGCCACTGTTAAGAGA
Dll4	AGGTGCCACTTCGGTTACAC
	GGGAGAGCAAATGGCTGAT
Foxn1	TGACGGAGCACTTCCCTTAC
	GACAGGTTATGGCGAACAGAA
Plet1	CATCCGTGAAAATGGAACAA
	TCACAGTTGGAGTCGTGTTTATG
Hes1	ACACCGGACAAACCAAAGA
	CGCCTCTTCTCCATGATAGG
Heyl	CTGAATTGCGACGACGATTGGT
	GCAAGACCTCAGCTTTCTCC
Ascl1	GCTCTCCTGGGAATGGACT
	CGTTGGCGAGAAACACTAAAG
FgfrIIIb	CCTGCGGAGACAGGTAACA
	CGGGGTGTTGGAGTTCAT
Il7	CTGCTGCAGTCCCAGTCAT
	TCAGTGGAGGAATTCCAAAGAT
Tbx1	GCTGTGGGACGAGTTCAATC
	ACGTGGGGAACATTCGTCT
Foxa1	GAACAGCTACTACGCGGACA
	CGGAGTTCATGTTGCTGACA
Hprt	TCCTCCTCAGACCGCTTTT
	CCTGGTTCATCATCGCTAATC
Hmbs	TCCCTGAAGGATGTGCCTAC
	AAGGGTTTTCCCGTTTGC
Ywhaz	CTTCCTGCAGCCAGAAGC
	GGTTTCCTCCAATCACTAG

Development • Supplementary information

Table S4. Primers used for genotyping

Gene name		Primer sequence (5'- 3')
Foxn1Cre	Forward	GACCAGGTTCGTTCACTCATGG
	Reverse	CCTTAGCGCCGTAAATCAATCG
RBPJ	Forward	GTTCTTAACCTGTTGGTCGGAAAC
	Wild type Reverse	GCTTGAGGCTTGATGTTCTGTATTGC
	Floxed allele Reverse	GGGCTGCTAAAGCGCATGCT
	Recombined allele Forward	CCTTGGTTTGTTGTTTGGGTT
	Recombined allele R	GTGGCTCTCAACTCCCAATCGT
sGFP	Forward	ACATGGTCCTGCTGGAGTTC
	Reverse	TCAGGTTCAGGGGGAGGT
R26NICD	Forward	AAAGTCGCTCTGAGTTGTTAT
	Transgenic R	GCGAAGAGTTTGTCCTCAACC
	Wild type R	GGAGCGGGAGAAATGGATATG
Venus	Forward	AAGTTCATCTGCACCACCG
	Reverse	TGCTCAGGTAGTGGTTGTCG
Ascl1	Wild type Forward	CTCCGGGAGCATGTCCCCAA
	Mutant Forward	GCAGCGCATCGCCTTCTATC
	Reverse	CCAGGACTCAATACGCAGGG

Development • Supplementary information

Table S5: Log2 RKPM for selected genes, from E14.5 Rbpj cKO PLET1+ and PLET1- and E14.5 wild-type PLET1+ and PLET1- samples. The data indicate a trend for downregulation of Notch 1, Notch 3, and Hey1 in E14.5 PLET1+ LOF versus WT samples, although this does not reach statistical significance. Full sample names in GEO (left to right across table): E14.5 WT+ rep1, E14.5 WT+ rep2, E14.5 WT+ rep3, E14.5 LOF+ rep1, E14.5 LOF+ rep2, E14.5 LOF+ rep3, E14.5 WT- rep1, E14.5 WT- rep2, E14.5 WT- rep3, E14.5 LOF- rep1, E14.5 LOF- rep2, E14.5 LOF- rep3.

**Developmental stage**		**E14.5**											
**PLET1 status**		**PLET1+**						**PLET1-**					
**Genotype & sample name**		**WT**			**LOF**			**WT**			**LOF**		
**Gene symbol**	**Ensembl ID**	**Rep1**	**Rep2**	**Rep3**	**Rep1**	**Rep2**	**Rep3**	**Rep1**	**Rep2**	**Rep3**	**Rep1**	**Rep2**	**Rep3**
Notch1	ENSMUSG00000026923	0.19	0.06	0.58	-8.69	-1.03	-1.74	-2.39	0.01	-2.30	-1.56	-1.25	-2.05
Notch2	ENSMUSG00000027878	4.91	3.40	4.02	6.43	3.29	3.44	4.78	3.64	3.46	5.20	3.27	3.68
Notch3	ENSMUSG00000038146	-0.16	-0.14	0.48	-8.20	-3.79	-5.55	-1.78	-0.45	-1.72	-4.82	-1.50	-2.28
Jag1	ENSMUSG00000027276	5.51	4.49	5.83	6.34	4.18	3.73	4.52	3.40	3.85	4.86	3.32	3.49
Hes1	ENSMUSG00000022528	4.32	4.10	4.43	2.97	4.10	4.49	4.50	4.63	4.82	3.88	4.06	3.41
Heyl	ENSMUSG00000032744	1.17	2.36	2.18	-5.52	0.18	-2.96	1.77	2.76	2.72	-2.39	2.03	1.25
Ascl1	ENSMUSG00000020052	-5.37	-0.65	0.54	0.37	-0.97	-3.54	-0.32	-0.61	-6.48	-5.83	-1.83	-6.48
Foxn1	ENSMUSG00000002057	4.89	5.95	4.94	2.64	5.51	4.58	5.06	6.30	6.58	4.42	5.82	5.52
Plet1	ENSMUSG00000032068	3.01	3.36	3.54	2.36	3.57	4.00	-5.85	1.77	-0.54	-5.85	0.67	-2.49

Development • Supplementary information

Table S6. Expression of selected known regulators of mTEC lineage development in wild type, RBPK cKO and NICD thymi at different developmental stages. Log2 RPKM of the RNAseq samples shown, presented as (A,B) means for each genotype and phenotype at E12.5 and E14.5 (C) data from the individual samples for each genotype and phenotype at E12.5 and (D) data from the individual samples for each genotype and phenotype at E14.5. WT, wild type. LOF, loss of function (*Foxn1^{Cre};Rbpj$^{fl/fl}$*). GOF, gain of function (*Foxn1^{Cre}*;*R26-stop-NICD-IRES-eGFP*).

Click here to Download Table S6

Data collection: Data collection was as in Fig 6. (E12.5 samples were PLET1^{+} TEC. E14.5 samples for WT and LOF were PLET1^{+} TEC (denoted WT-5+ etc) and PLET1^{-} TEC (denoted WT-5- etc), and for GOF were PLET1^{+} TEC. Note that the RNAseq data presented here were obtained from 100 TEC per sample from embryos at developmental stages, as described in Materials and Methods. The E12.5 and E14.5 samples were each obtained from two separate litters, on two separate days for each timepoint. The sample-to-sample variation at each timepoint is relatively high for genes expressed at low levels, likely due to factors including differences in the precise developmental age of the embryos from which cells were obtained for the different samples; and 'drop out' of low frequency cell populations and signal from low expression level genes. Additionally, this model deletes after some mTEC have already emerged which compounds this analysis as some differentiating mTEC are present in the LOF samples. For these reasons, we have been very cautious in interpreting these data and refer the reader to the full data table for consideration of any trends observed.

Development • Supplementary information

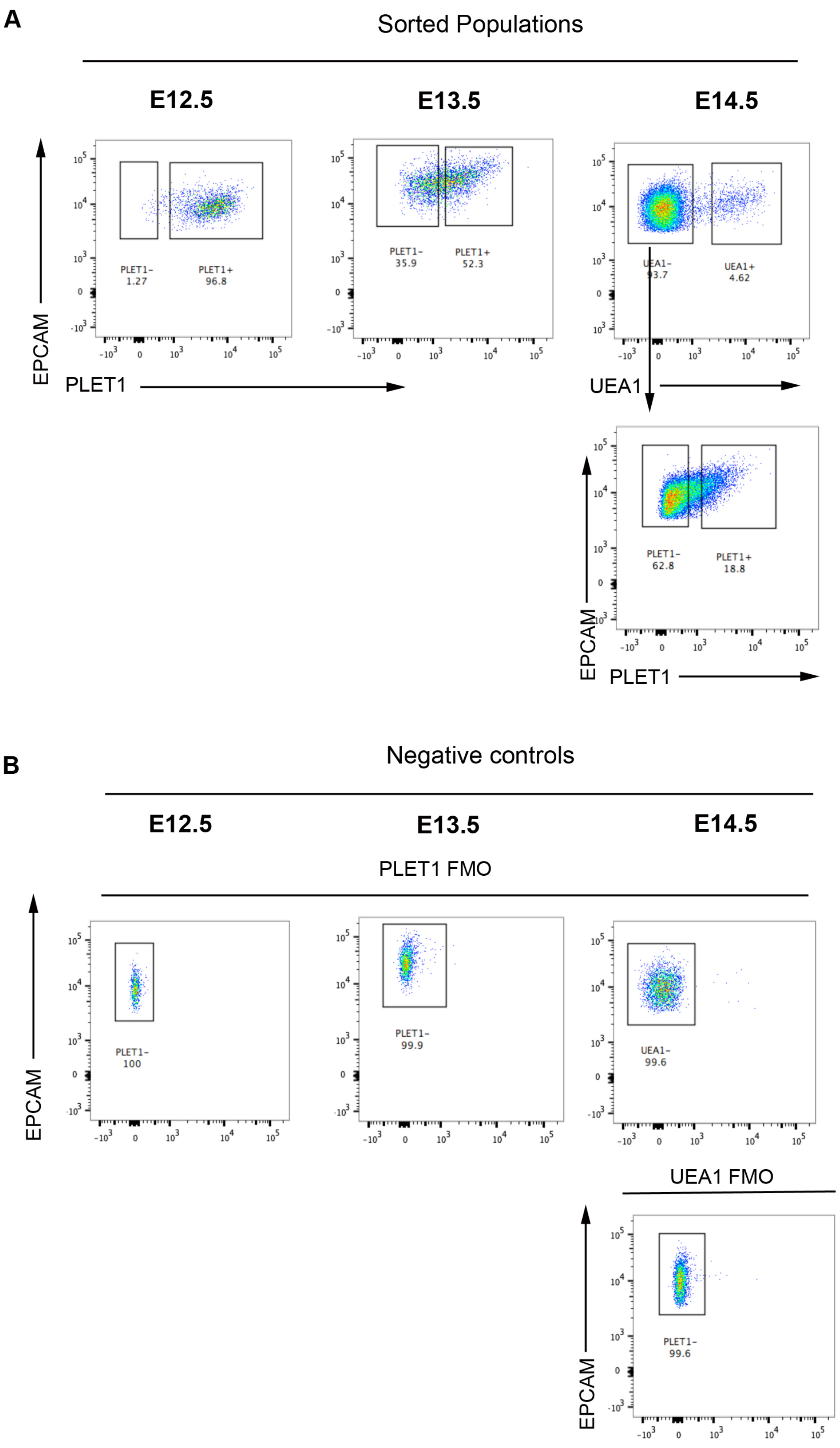

Figure S1. Sorting strategy for fetal TEC populations, related to Figs 1, 6 and Supplementary Figs. 8, 9 and 10. All cells were pre-gated on DAPI$^-$Lineage$^-$EPCAM$^+$ epithelial population. PLET1$^+$ and UEA1$^+$ populations were determined by comparing with FMO controls. Lineage panel: CD45, CD31, TER119, CD11c.

Development • Supplementary information

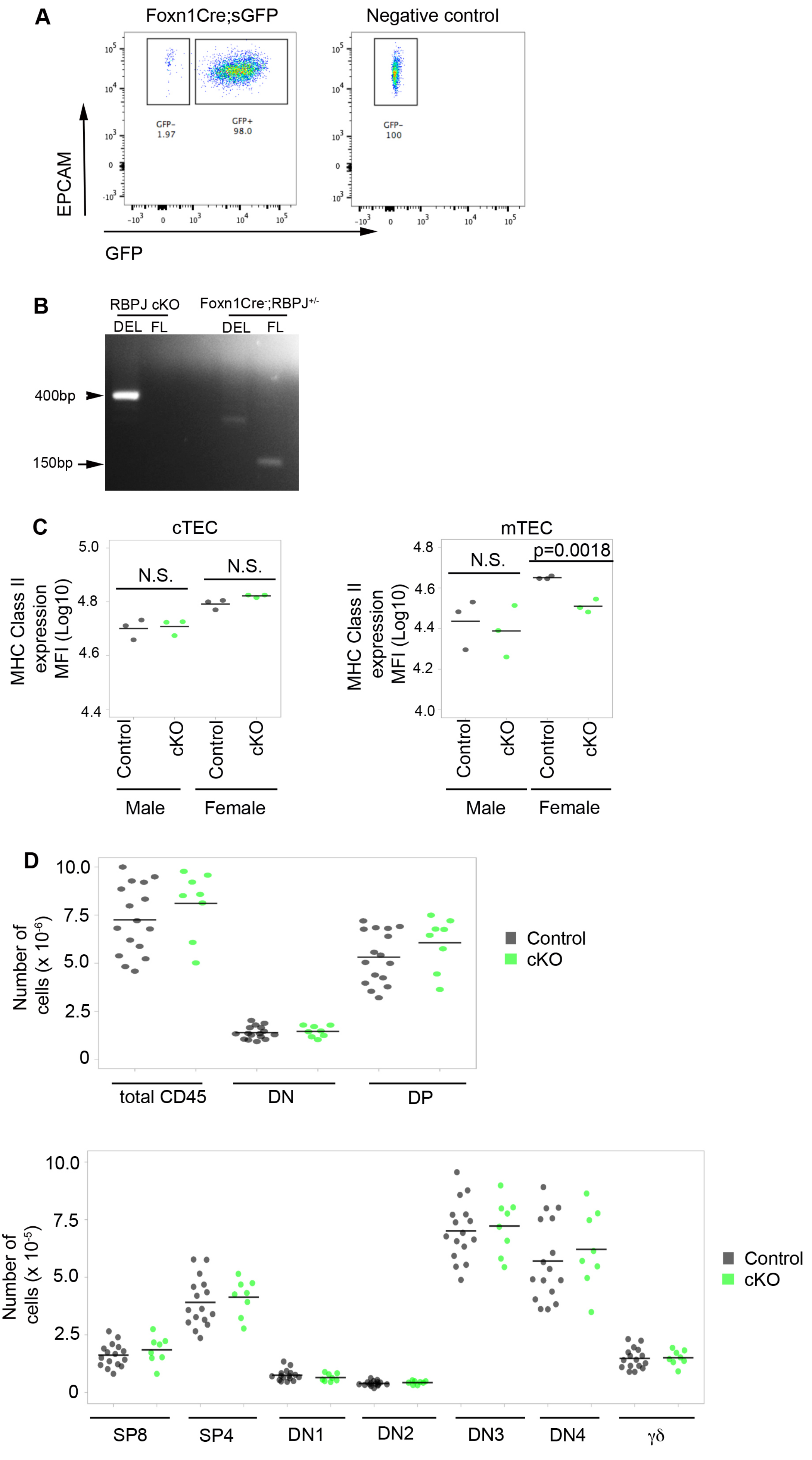

Development • Supplementary information

Figure S2. Characterization of recombination efficiency of the RBPJ cKO, MHC Class II expression and thymocyte subset distribution in RBPJ cKO thymi, related to Figures 2**.** (A) The recombination efficiency of *Foxn1*^{Cre} was tested using a silent GFP strain. By E14.5, most TECs had activated GFP, demonstrating a history of Cre activity in these cells. (B) Genomic PCR showing the genotype of flow cytometrically sorted total TECs in 4 week-old mice. The deleted (DEL) (400bp) but not the floxed (FL) allele (150bp) of Rbpj was detectable in the RBPJ cKO TECs. (C) Median fluorescence intensity for MHC Class II expression in cTECs and mTECs from two-week-old male and female mice of the genotypes shown. Data are shown as Log_{10} transformed due to population distribution. p-values: male cTEC, 0.8023; female cTEC, 0.0552; male mTEC, 0.6624; female mTEC, 0.0018. (D) Thymocyte subset profile in E18.5 *Rbpj* cKO and littermate control thymi. No differences were observed between cKO and controls. ***Data collection:*** (A,B) n>5 mice. (C) n=3 cKO and 3 littermate control mice for male and female; the mice used were the same mice as in Figure 2. (D) n=8 cKO and 16 littermate control embryos, collected from two separate litters. All samples were analyzed and genotype was retrospectively confirmed.

Statistics: p values in pairwise comparisons were calculated with two-tailed t-test.

Development • Supplementary information

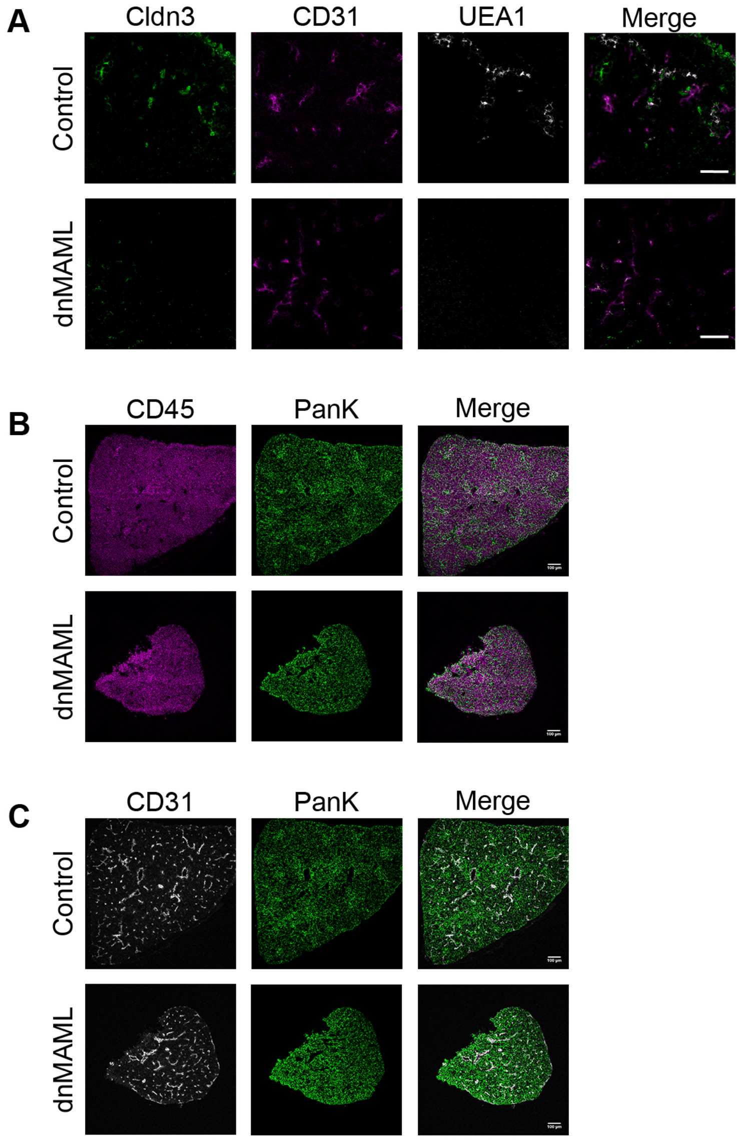

Figure S3. Fetal dnMAML thymi are populated with thymocytes and endothelial cells, related to Figure 4**.** (A) Images showing E14.5 control (aged-matched *Foxa2*^{T2iCre}*;Gt(ROSA)26So*$^{rtm1(EYFP)Cos}$) and dnMAML sections stained with Claudin3 (CLDN3), endothelial marker CD31 and mTEC marker UEA1. Note that certain weakly stained CLDN3^{+} cells co-localized with endothelial marker CD31. Scale bar=50µm. (B-C) E16.5 control and dnMAML sections stained with thymocyte marker CD45 (B), CD31 (C) and counterstained with epithelial marker Pan-keratin (PanK). Scale bar=100µm. The size of E16.5 dnMAML sections are typically smaller than controls.

Data collection: as for Figure 4.

Supplementary Figure 4

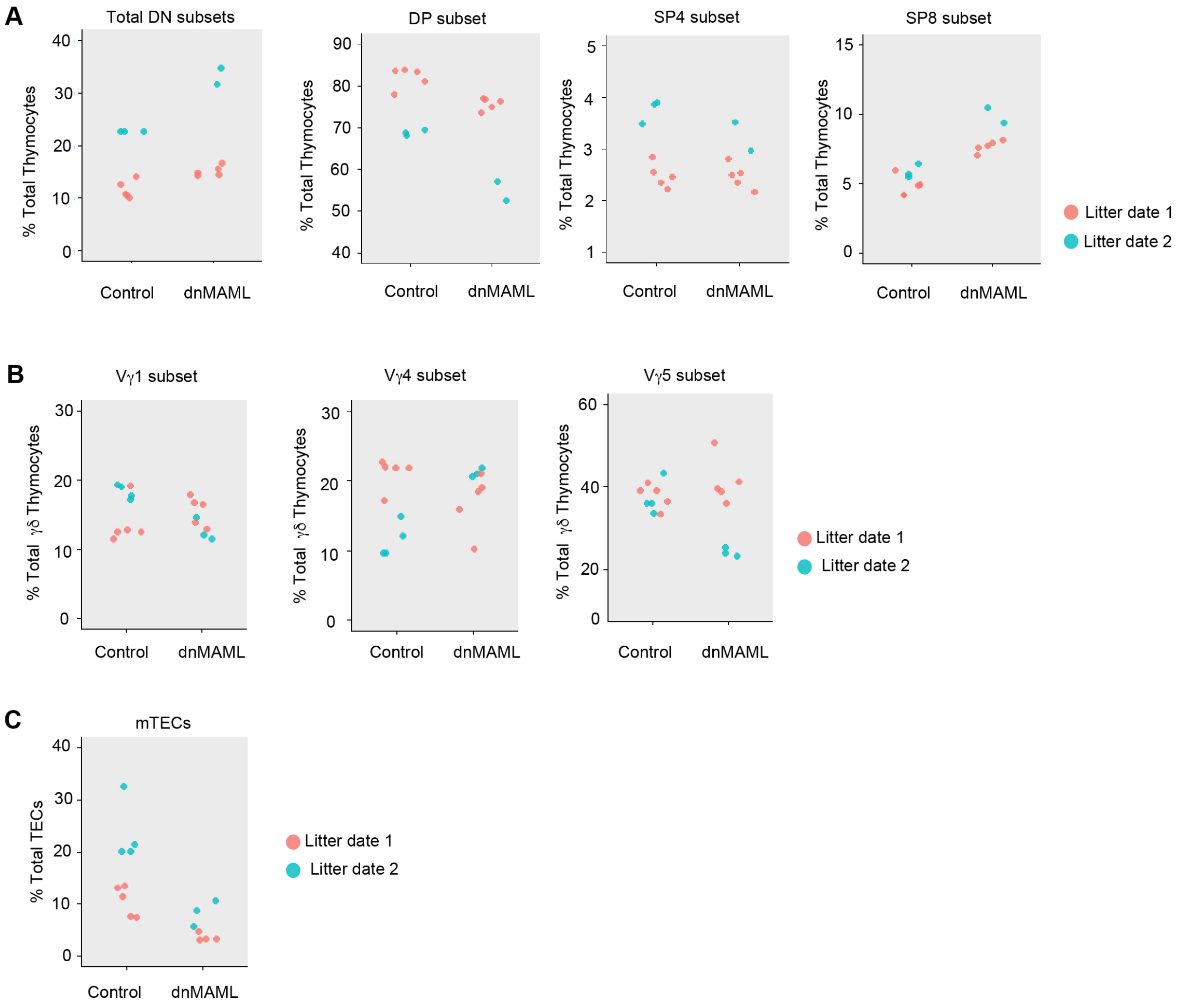

Figure S4. Thymocyte development in Fetal dnMAML thymi, related to Figure 4. Thymi from individual E17.5 embryos of the genotypes shown, collected on two different analysis dates, were analyzed for (A) DN (left), DP (middle left), $CD4^+$ SP (middle right) and $CD8^+$ SP (right) thymocytes; (B) Vγ1, Vγ4 and Vγ5 thymocyte subsets; and (C) $UEA1^+$ mTEC. Plots show summarized percentages of flow cytometric analysis. Each data point represents an individual embryonic thymus.

Data collection: Litter date 1: 2 litters each of control and dnMAML embryos were taken at E17.5, containing 21 and 19 embryos respectively. The analyses shown in panels (A-C) were set up independently, each using five control and five dnMAML thymi. Controls were *Foxa2*^{T2iCre} embryos. One extreme dnMAML outlier was excluded from the TEC summary plot shown in (C). This embryo exhibited poor Cre-mediated activation of dnMAML, as reported by eGFP expression. No other data were excluded. The panels used for flow cytometric analysis were: A. Viability (Zombie

Development • Supplementary information

NIR dye), Lineage (FITC; CD11b, CD11c, B220, GR-1, NK1.1, TER119, EpCAM, CD31) CD4 (PE), CD8 (APC), CD3ε (BB700), TCRβ (PE/Cy7), CD62L (BV605), MHCI (BV510), CD69 (BV421). B. Viability (Zombie NIR dye), Pan γδ TCR (PE/Cy7), Vγ1 (PerCp/Cy5.5), Vγ1 + Vγ2 (PE), Vγ4 (APC), Vγ5 (BV605). C. Viability (DAPI), Lineage (PerCP/Cy5.5; CD45, CD31, CD11b, TER119), EpCAM (PE), CD205 (PE/Cy7), UEA1-biotin (streptavidin-BV605), PLET1 (anti-rat AF647), MHCII (APC/Fire).

Litter date 2: One litter each of control and dnMAML embryos were taken at E17.5, containing eight and six embryos respectively. Four control and three dnMAML embryos were analyzed for TEC and γδ thymocyte subset distribution. A different set of four control and three dnMAML embryos were analysed for total thymocyte progression. Two extreme outliers (1 control and 1 dnMAML) were excluded from the thymocyte development summary plots shown in (A). The panels used for flow cytometric analysis were as follows. TEC panel: EpCAM (PE), CD205 (PECy7), UEA1-biotin (BV650-streptavidin), PLET1 (1D4, neat, Goat α-rat Alexafluor647), Lin (CD45, CD31, CD11c, TER119), DAPI, GFP. Thymocyte panel: CD45 (APCeFluor780), CD4 (BV650), CD8 (PerCP-Cy5.5), CD44 (PECy7), CD25 (PE), DAPI, GFP. γδ thymocyte panel: TCRγδ (PECy7), Vγ5 (BV605), Vγ1 (PerCP-Cy5.5), Vγ4 (APC), CD45 (APCeFluor780) DAPI, GFP.

Development • Supplementary information

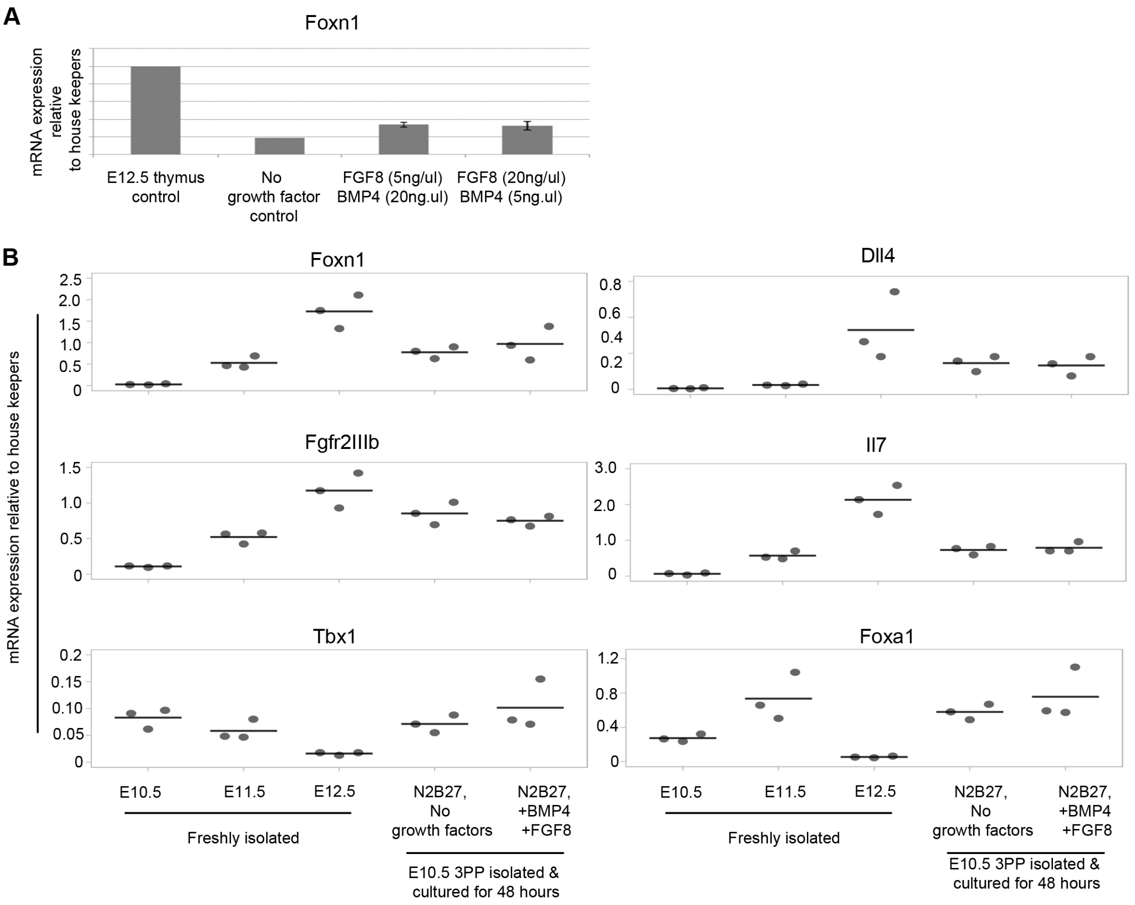

Figure S5. Culture of E105 3PP explants in TEPC conditions mimics key features of thymic primordium development, related to Figure 4, and Supplementary Figs 5 and 6. (A) Expression of *Foxn1* in whole E10.5 3PP explants after culture in N2B27 medium supplemented with the growth factors shown for 2 days. The combination of FGF8 and BMP4 increases *Foxn1* mRNA expression. (B) Expression of the genes shown in EPCAM$^+$PLET1$^+$ TEC recovered from E10.5 3PP explants after 2 days of culture in N2B27 ± FGF8 and BMP4 (with growth factors = TEPC medium), versus purified E10.5 and E11.5 EPCAM$^+$PLET1$^+$ 3PP cells and E12.5 EPCAM$^+$PLET1$^+$ *ex vivo* TEC. Regulation of key genes occurs with similar dynamics in culture as *in vivo*. Note that in the explant culture, the parathyroid primordium cannot separate from the thymus primordium, likely accounting for the higher levels of *Tbx1* seen in the explant cultures compared to E12.5 thymic primordium (parathyroid primordium cells also express PLET1). Also note that E10.5 3PP have not yet been colonized by T cell progenitors [39] and therefore the explants did not contain thymocytes.

Data collection: (A) n=3. (B) B, data from 3 independent biological replicates are shown for each timepoint or condition. Each data point represents relative expression levels normalized to the geometric mean of three housekeepers, obtained from technical triplicates.

Development • Supplementary information

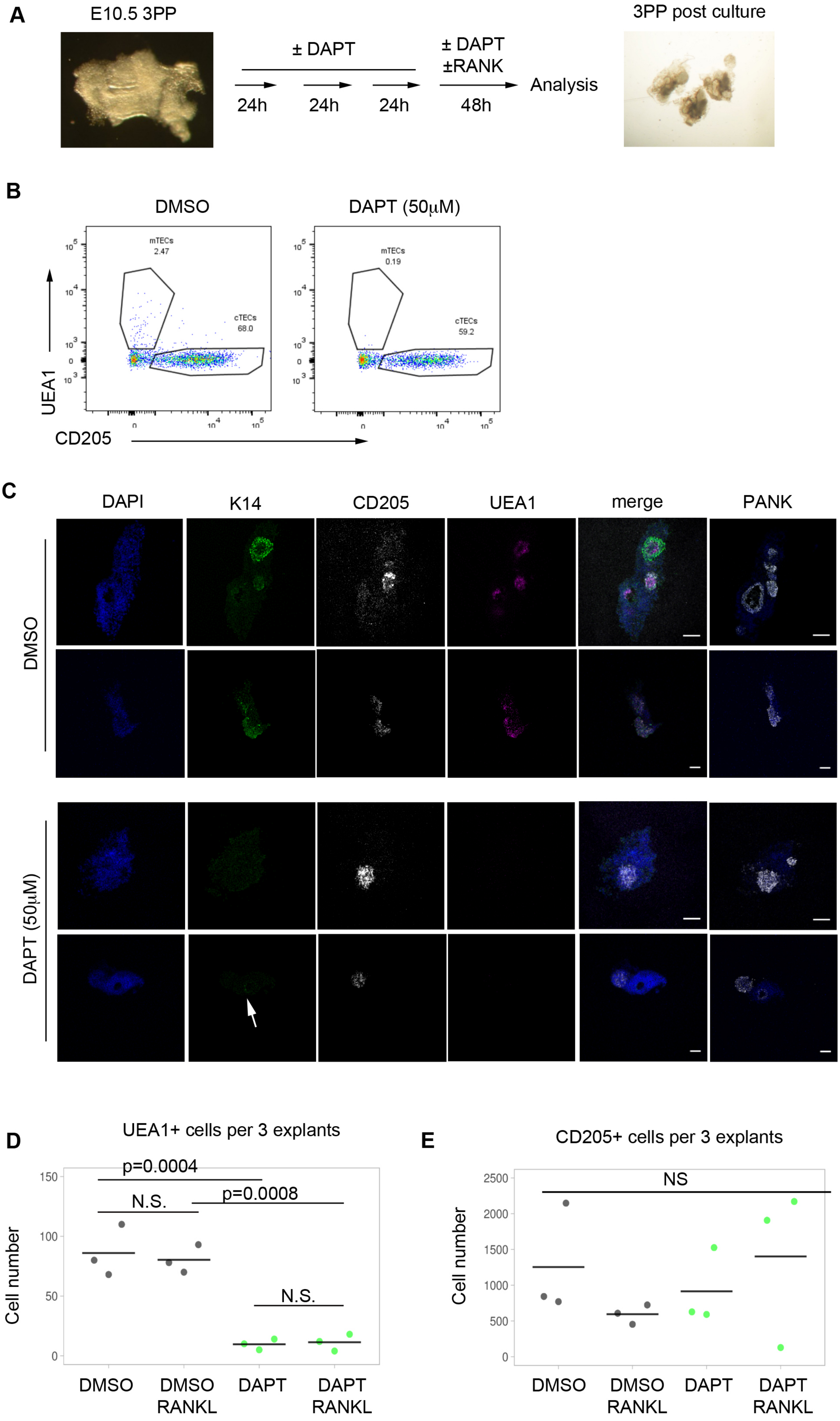

Development • Supplementary information

Figure S6. Notch signalling is required for mTEC specification, related to Figure 4. (A) Image shows an example of 3PP dissected from a ~35-somite stage embryo upon seeding into TEPC medium. Experimental scheme is shown on the right. (B) (Left) Representative plots showing UEA1$^+$ mTECs and CD205$^+$ cTECs emerging in E10.5 3PP explants after 5 days in culture in the presence or absence of 50μM DAPT throughout the culture period. (C) Representative images of explants stained for mTEC markers K14 and UEA1, and cTEC marker CD205. The adjacent sections were stained for PanK to demonstrate presence of epithelial cells. Arrow: K14$^+$ cells in DAPT-treated explants, localized to what appears to be the remnant of 3PP cells lining the lumen. Scale bars=100μm. (D, E) Quantitation of the number of UEA1$^+$ (D) and CD205$^+$ (E) cells after culture. Each data point is the total of three explants. n=3 independent biological replicates. Of note is that the freshly dissected 3PP explants contained parathyroid-fated cells and in some cases epithelial cells from adjacent pouches, as well as thymus-fated cells (Supplementary Fig. 6A). Therefore, when quantifying the effects of DAPT we calculated the number of cells expressing mTEC and cTEC-associated markers (UEA1 and CD205 respectively), rather than using EPCAM$^+$ total epithelial cells.

Data collection: (B-E) Wildtype embryos from several E10.5 litters were used to set up each experiment. For each experiment, three 3rd pharyngeal pouch tissue pieces were cultured for each condition. Cultures were established and analysed on three different days. (B,D,E) n=3 independent experiments. Each data point is the sum of all three explants cultured for one independent replicate. (C) Representative images of n=4 independent experiments.

Statistics: p values were calculated using Two-tailed one-way ANOVA.

Development • Supplementary information

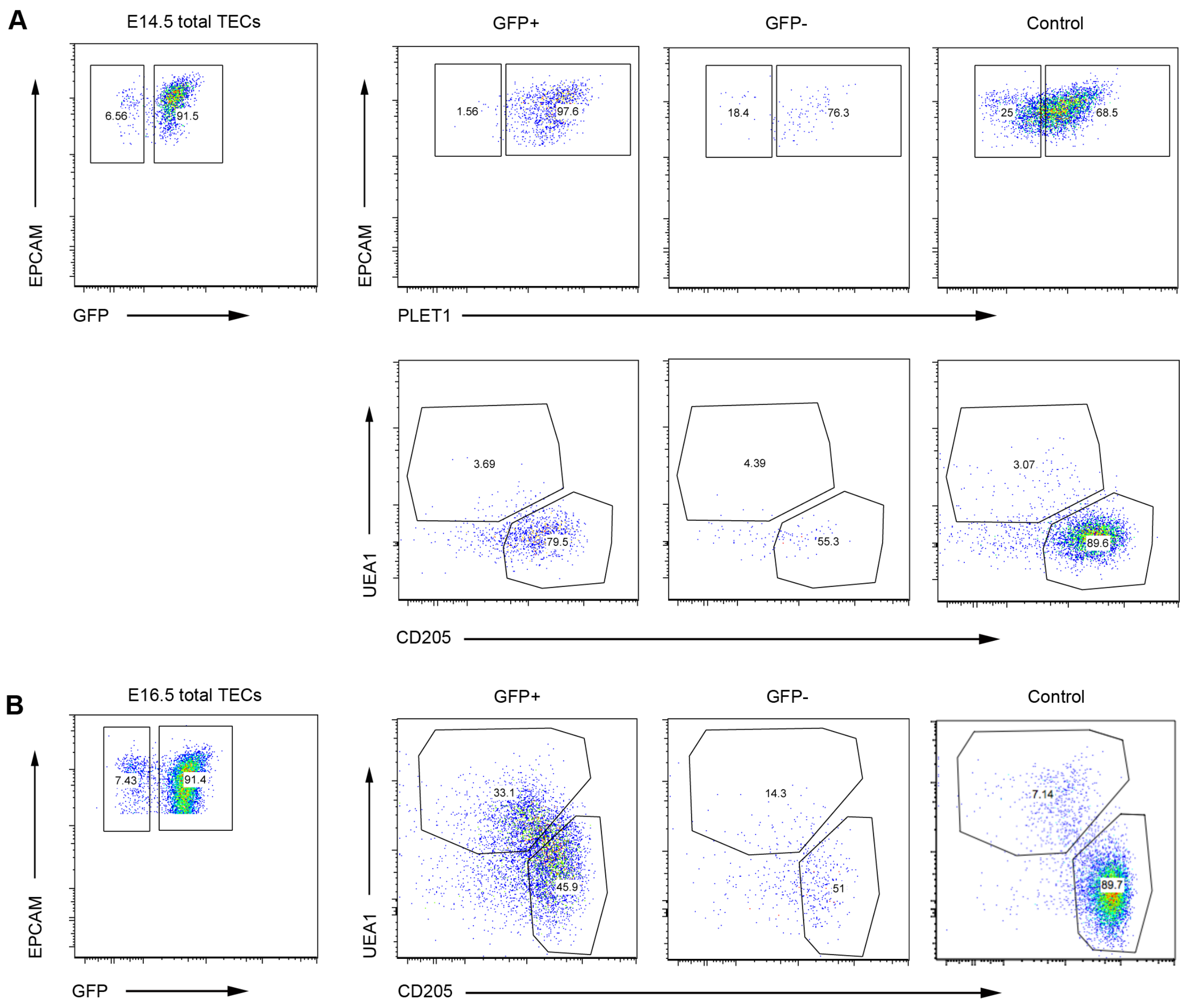

Figure S7. The effect of NICD upregulation in TEC is cell autonomous, as revealed by mosaic deletion of RBPJ in Foxn1^{Cre};R26-stop-NICD-IRES-eGFP (NICD) fetal thymi, related to Figure 5. In this model, GFP and NICD should be induced simultaneously by *Foxn1^{Cre}*, however we observed mosaic expression of GFP in the NICD thymi, indicating the presence of a small proportion of cells in which Cre-mediated activation of NICD had not occurred. Comparison of the GFP^{+} and GFP^{-} TEC populations within the same E14.5 thymi (A) revealed that the GFP^{+} TEC were almost universally PLET1^{+}, whereas the GFP- subset PLET1 profile resembled that of the control thymi (see also Fig. 6C). The UEA1/CD205 subset profile was similar in the GFP^{+} and GFP^{-} subsets at E14.5. This confirmed that the altered PLET1 levels in GFP^{+} cells resulted from high Notch activity and further indicated that this effect was cell autonomous. At E16.5 (B), a higher percentage of GFP^{+} than GFP^{-} was UEA1^{+}, but the proportion of UEA1^{+} TEC in the GFP^{-} subset was higher than controls.

Data collection: as for Figure 6.

Development • Supplementary information

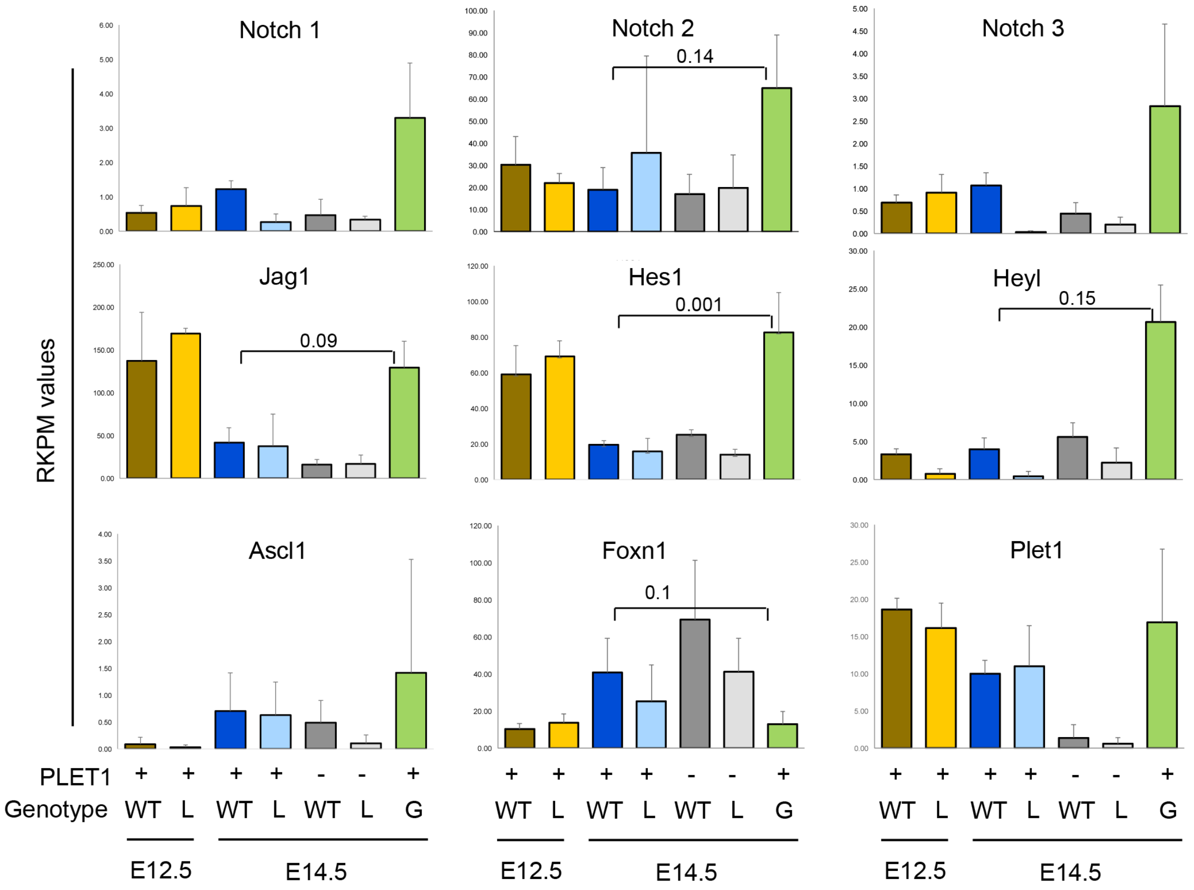

Figure S8, Expression levels of Notch family genes in Rbpj cKO, NICD and wild type fetal TEC datasets, related to Figure 6 and Supplementary Figures 8 and 9. Overview of the average expression levels (RPKM values) of Notch signalling target genes for the E12.5 PLET1$^+$ *Rbpj* cKO and control (yellow and brown bars); E14.5 PLET1$^+$ *Rbpj* cKO and control (light blue, dark blue respectively); E14.5 PLET1$^-$ *Rbpj* cKO and control (light grey, dark grey respectively); and E14.5 PLET1$^+$ NICD samples (green). No consistent differences were found between the PLET1$^+$ *Rbpj* cKO and control samples at E12.5. A more consistent trend for down-regulation of the Notch target genes was observed in E14.5 PLET1$^+$ *Rbpj* cKO TEC versus WT controls; this trend was absent when comparing the E14.5 PLET1$^-$ *Rbpj* cKO and WT control samples. FDR for E14.5 PLET1$^+$ NICD versus E14.5 WT samples is shown where significant. ***Data collection:*** as for Figure 7.

Statistics: Error bars show mean±SD.

Development • Supplementary information

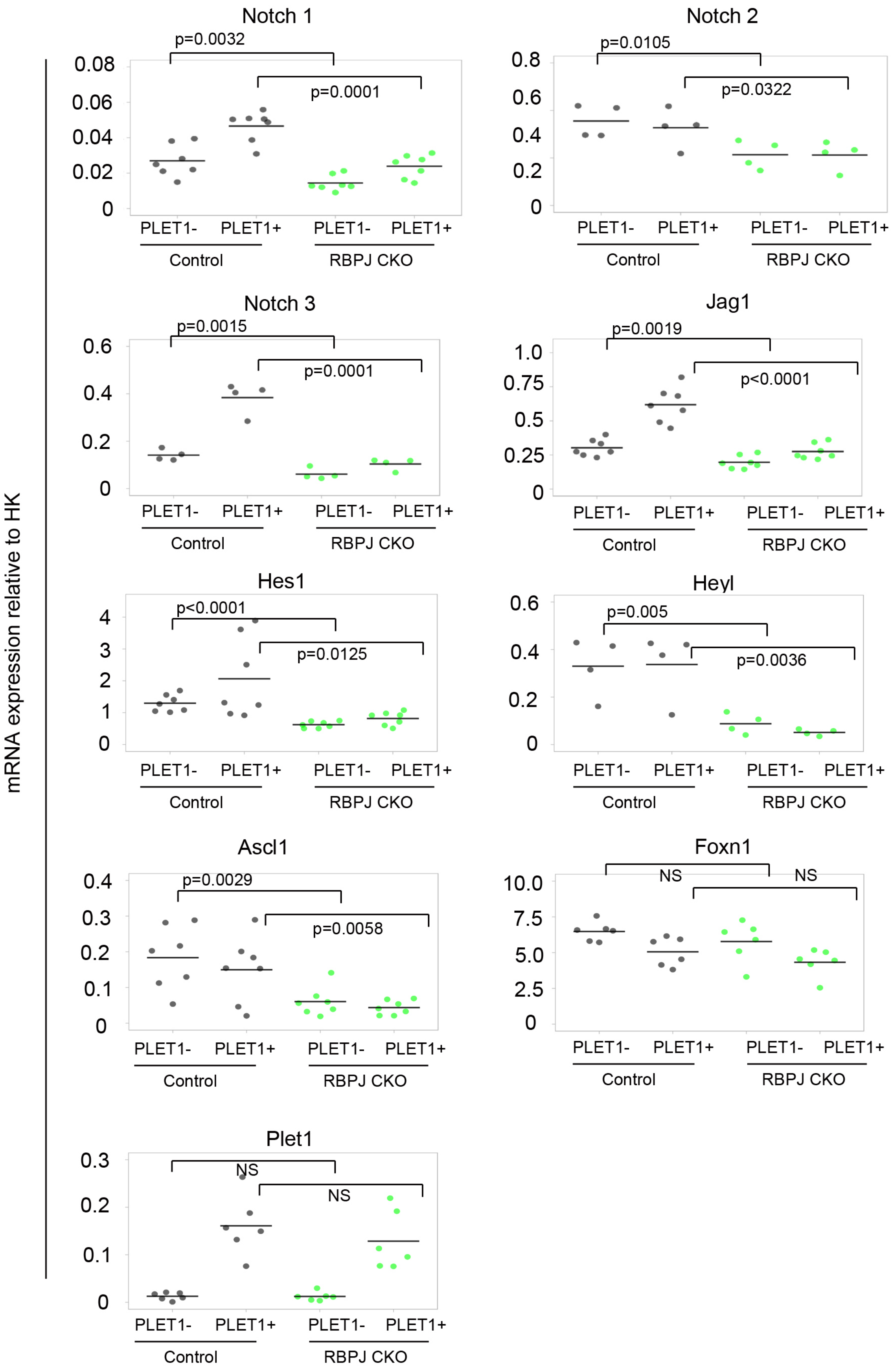

Figure S9. Loss of Notch signalling in TEC leads to loss of expression of Notch family genes, indicating a positive feedback loop, Related to Figure 6. mRNA expression levels in E14.5 control and *Rbpj* cKO TECs, determined by RT-qPCR analysis of sorted

Development • Supplementary information

cell populations of the phenotypes and genotypes shown. Each data point represents relative expression levels normalized to the geometric mean of three housekeepers, obtained from technical triplicates.

Data collection: Notch1, n=7; Notch2, n=4; Notch3, n=4; Jag1, n=7; Hes1, n=7; Heyl, n=4; Ascl1, n=7; Foxn1, n=6; Plet1, n=6, where n is an independent embryo of the genotype shown.

Statistics: Two-tailed unpaired t-test, comparing (1) PLET1^{+} control vs cKO, and (2) PLET1^{-} control vs cKO.

Development • Supplementary information

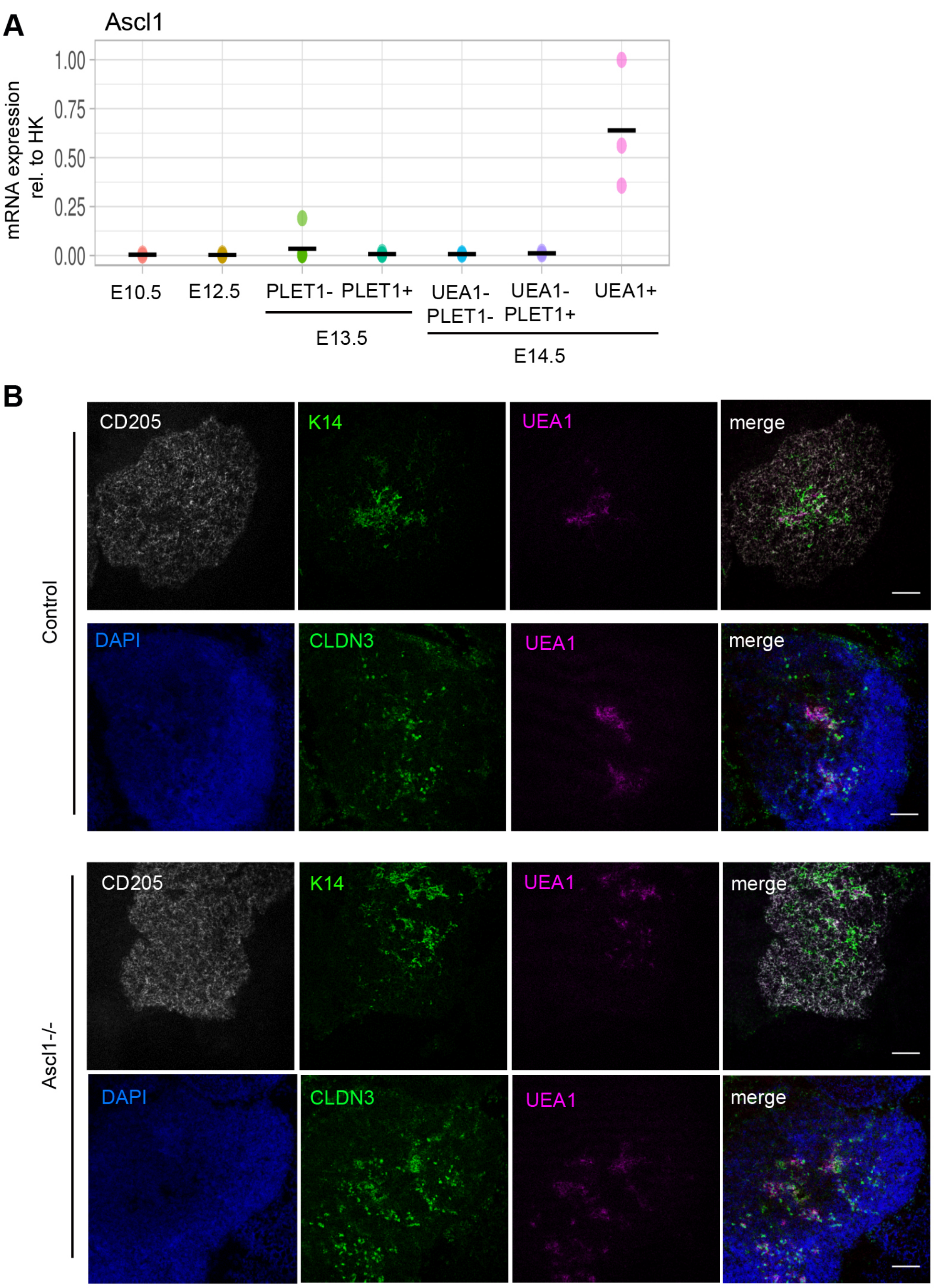

Figure S10 ASCL1 does not regulate mTEC specification, related to Figure 6. (A) *Ascl1* mRNA expression in fetal TEC populations. Strong upregulation in observed in E14.5 mTECs, concomitant with mTEC emergence and expansion. Each data point represents relative expression levels normalized to the geometric mean of three housekeepers, obtained from technical triplicates. (B) Images show cryosections of E17.5 control and *Ascl1* null mutant thymi stained with the markers shown. No difference in marker expression was observed. Scale bar=100µm.

Data collection: (A) n=6 independent biological replicates. (B) Representative of 2 independent embryos.

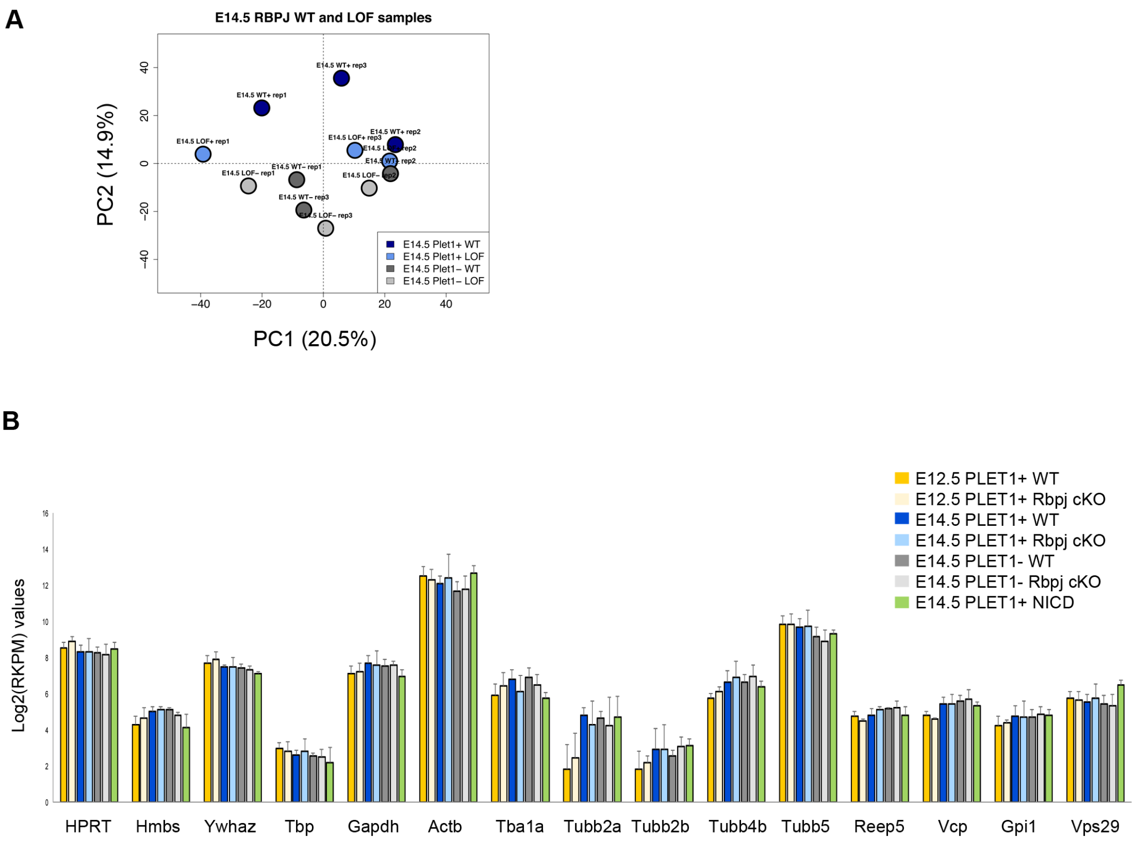

Figure S11, Principal component analysis of E14.5 wild type and Rbpj cKO PLET1$^+$ and PLET1$^-$ TEC, and expression of housekeeping genes, related to Figure 6 and Materials and Methods. (A) Principal component analysis of the 2000 most variable genes of the E14.5 wild type and *Rbpj* cKO group showed a separation of the E14.5 PLET1$^+$ wild type samples (dark blue) against the rest (E14.5 *Rbpj* cKO and PLET1$^-$ wild type samples) on PC2. PC1 is representative of the strong biological variation that exists among replicates since samples cluster per their biological sample number (e.g. E14.5 LOF+ rep1, E14.5 LOF- rep1). (B) Overview of the average expression levels (RPKM values) of housekeeping genes over the E12.5 PLET1$^+$ *Rbpj* cKO (yellow bars) and control (brown bars), the E14.5 PLET1$^+$ *Rbpj* cKO and control (light blue, dark blue respectively), the E14.5 PLET1$^-$ *Rbpj* cKO and control (light grey, dark grey respectively) and the E14.5 PLET1$^+$ NICD samples (green). The expression profile of housekeeping genes among all samples does not show strong effects towards any particular groups (see Figure 8B), indicating that it is biological differences than strong batch effects that are driving the separation of the groups.

Data collection: as for Figure 7.

Statistics: Error bars show mean±SD.

Development • Supplementary information
